# Predicting dynamic cellular protein–RNA interactions by deep learning using in vivo RNA structures

**DOI:** 10.1038/s41422-021-00476-y

**Published:** 2021-02-23

**Authors:** Lei Sun, Kui Xu, Wenze Huang, Yucheng T. Yang, Pan Li, Lei Tang, Tuanlin Xiong, Qiangfeng Cliff Zhang

**Affiliations:** 1 MOE Key Laboratory of Bioinformatics, Beijing Advanced Innovation Center for Structural Biology and Frontier Research Center for Biological Structure, Center for Synthetic and Systems Biology, School of Life Sciences, Tsinghua University, Beijing 100084, China; 2 Tsinghua-Peking Center for Life Sciences, Beijing 100084, China; 3 Program in Computational Biology and Bioinformatics, Yale University, New Haven, CT 06520 USA; 4 Department of Molecular Biophysics and Biochemistry, Yale University, New Haven, CT 06520 USA

**Keywords:** Molecular biology, Bioinformatics, Mechanisms of disease

## Abstract

Interactions with RNA-binding proteins (RBPs) are integral to RNA function and cellular regulation, and dynamically reflect specific cellular conditions. However, presently available tools for predicting RBP–RNA interactions employ RNA sequence and/or predicted RNA structures, and therefore do not capture their condition-dependent nature. Here, after profiling transcriptome-wide in vivo RNA secondary structures in seven cell types, we developed PrismNet, a deep learning tool that integrates experimental in vivo RNA structure data and RBP binding data for matched cells to accurately predict dynamic RBP binding in various cellular conditions. PrismNet results for 168 RBPs support its utility for both understanding CLIP-seq results and largely extending such interaction data to accurately analyze additional cell types. Further, PrismNet employs an “attention” strategy to computationally identify exact RBP-binding nucleotides, and we discovered enrichment among dynamic RBP-binding sites for structure-changing variants (riboSNitches), which can link genetic diseases with dysregulated RBP bindings. Our rich profiling data and deep learning-based prediction tool provide access to a previously inaccessible layer of cell-type-specific RBP–RNA interactions, with clear utility for understanding and treating human diseases.

## Introduction

RNA binding proteins (RBPs) play essential roles in regulating the transcription, metabolism, and translation of cellular RNAs.^1–4^ Determining RBP binding profiles in different conditions and elucidating their detailed regulatory mechanisms are critical for understanding their functions. However, given the sheer number of RBPs that account for close to 10% of the human proteome,^5,6^ establishing links between RBPs and their targets has been an enormous challenge. To address this question, many high-throughput technologies have been developed to profile and predict RBP binding. Assays such as systematic evolution of ligands by exponential selection (SELEX), RNAcompete, and RNA Bind-n-Seq can characterize the sequence preferences of RBPs in vitro,^7–9^ and methods like RNA immunoprecipitation (RIP) and UV crosslinking followed by immunoprecipitation (CLIP) and sequencing can identify RBP binding sites in vivo.^10–13^

In addition to the methods of direct measuring, other approaches have been developed to model and predict RBP binding. Traditionally, position-weight-matrices have been used to describe RBP binding determinants and to predict RBP binding targets from RNA sequences.^14^ Machine learning methods that integrate different types of information also have been developed to more accurately characterize the binding pattern of RBPs.^15–17^ More recently, deep learning^18^ approaches have been successfully applied to model protein–RNA interactions and predict RBP binding sites.^19–24^ For example, DeepBind was developed to learn RBP binding preferences from RNAcompete data using a deep neural network.^20^

Although these learning methods successfully capture RBP binding preferences of primary sequence, their prediction accuracies under different physiological states are limited because the RNA sequence is independent of in vivo conditions. Over the years, several methods have been developed to include RNA structural features of RBP targets in their modeling, but these structures were based on computational prediction rather than in vivo analysis.^15–17,24^ Although RNA structure can be predicted from sequence with some accuracy,^25,26^ the predictions do not reflect the dynamic regulations by cellular trans-factors and usually show substantial differences from in vivo structures.^27,28^ Thus, in vivo RNA structure data are essential for accurate modeling and predictions of protein-RNA interactions in physiologically relevant contexts.

Here, we bridge this knowledge gap by determining transcriptome-wide RNA secondary structures in multiple cell types. We then integrate this experimentally-derived structure information in the construction of a deep discriminative neural network Protein-RNA Interaction by Structure-informed Modeling using deep neural NETwork (PrismNet) that accurately models and predicts RBP targets in vivo. We apply PrismNet to predict how genomic variants affect RBP binding, especially in the context of human diseases. Specifically, we focused on single nucleotide variants that disrupt RNA structure (riboSNitches) and are often associated with human disease, and discovered that riboSNitches are enriched in dynamic, cell-type-specific RBP binding sites.

## Results

### RNA structuromes in different cell types reveal the prevalence of structurally variable sites and their association with dynamic RBP binding

**Fig. 1:**
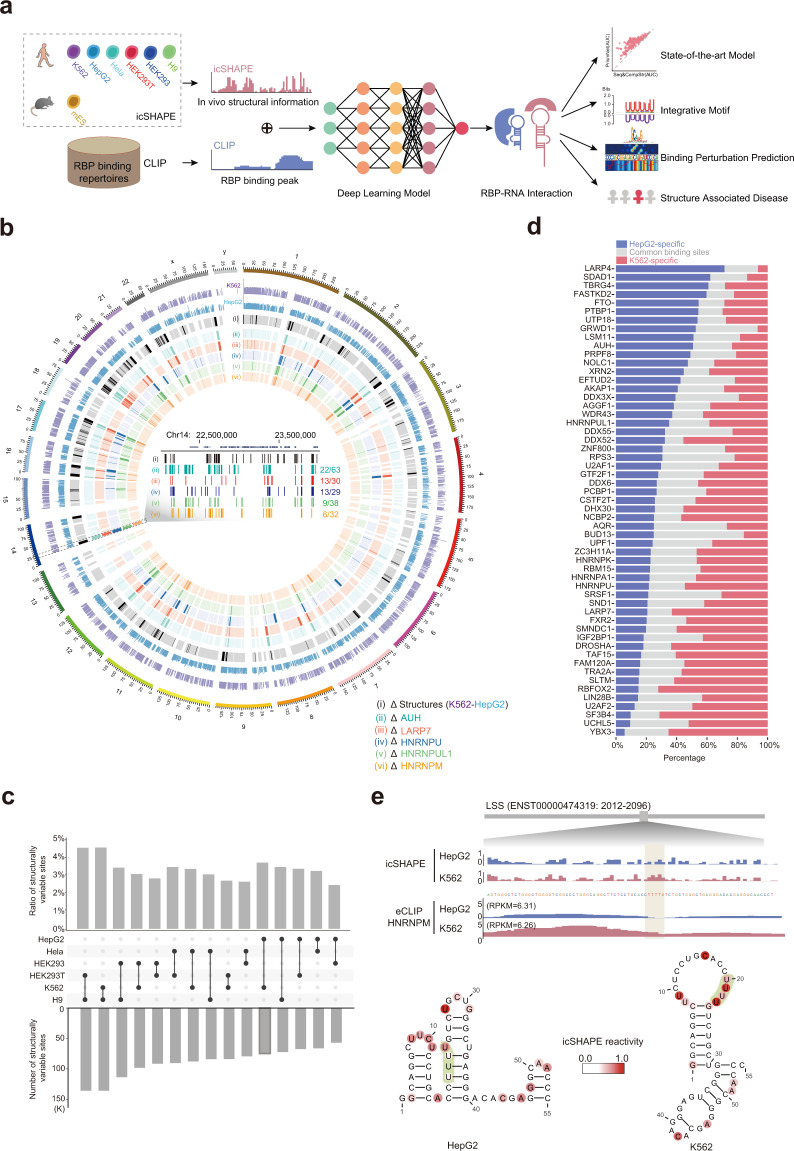
Association between RNA structural variations and dynamic RBP bindings can be used to predict RBP bindings in varying cellular contexts. **a** Integrative modeling and prediction of RBP bindings by PrismNet using in vivo RNA structure information and RBP binding sites from matched types of cells. PrismNet can be used to dissect and predict the perturbation effects of disease-associated genetic variations on RBP binding. **b** Circos plot showing the relationship between RNA structural variations (ΔStructures) and dynamic RBP binding sites (ΔRBP) in HepG2 and K562 cells. The transcripts in the region chr14:22,000,000–24,000,000 are magnified to illustrate that dynamic RBP binding sites are overlapped with RNA structural variations. Numbers show the fraction of overlapped RBP binding sites for the indicated RBPs (e.g., 22/63, 22 is the number of overlaps between ΔRBP and ΔStructures. 63 is the number of dynamic RBP binding sites (ΔRBP) in pairwise comparisons between cell lines). **c** Bar plot of the ratio and the number of RNA structurally variable sites for all pairwise comparisons of the six indicated human cell lines. **d** Stacked bar plots of the percentage of cell type-specific and common RBP binding sites in two cell lines from eCLIP datasets: HepG2-specific (blue), K562-specific (red), as well as common binding sites (gray). **e** RNA structural and HNRNPM binding profiles in HepG2 and K562 cell lines. Top, icSHAPE scores in the two cell lines for the *LSS* mRNA transcript; Middle, binding site of HNRNPM on the *LSS* mRNA transcript (eCLIP); Bottom, RNA structural models of the HNRNPM binding sites on the *LSS* mRNA transcript in the two cell lines. Models were constructed using RNAshapes with icSHAPE score constraints. Green dashed lines indicate the known HNRNPM poly-U binding motif.

RNA structure is flexible, and this feature plays an instrumental role in determining the varying protein–RNA interactions in different cellular conditions.^2,29,30^ In vivo click selective 2′-hydroxyl acylation and profiling experiment (icSHAPE) technology can be used to determine the RNA structural dynamic across the whole transcriptome.^27^ To characterize relationships between RNA structure and RBP binding globally, we generated a comprehensive resource of RNA secondary structures determined by icSHAPE in seven cell types: K562, HepG2, HEK293, HEK 293T, HeLa, H9, and mES cells (Fig. 1a; Supplementary information, Fig. S1a). These cell lines were selected for our structure profiling experiments because they all have rich RBP binding data from CLIP-seq experiments (Supplementary information, Fig. S1a), thereby enabling later integrative structural-and-interaction modeling of RBPs in a matched cellular context.

On average, we obtained at least 200 million usable reads for each library of two biological replicates after quality control, totaling 4.4 billion reads (Supplementary information, Table S1). We determined RNA secondary structures of the transcripts using icSHAPE-pipe.^31^ Our data achieved high coverage of the global transcriptomes (> 50,000 transcripts in human; >30,000 transcripts in mouse) as well as high quality (RPKM Pearson correlation coefficient > 0.97 between replicates) (Supplementary information, Fig. S1a, b). For example, our icSHAPE profiling data on 18S rRNA from different human cell lines were highly consistent (Supplementary information, Fig. S1c) and agreed well with known 18S secondary structures from crystal structures (Supplementary information, Fig. S1d, e).

Previously, we found that although RNA structure is relatively stable across different subcellular locations, there are a large number of structurally variable sites, many of which are hotspots for post-transcriptional regulation processes including RBP binding and RNA modification.^32^ We found that this is also true when comparing RNA structures across different cell lines, i.e., most of the RNA structures are stable across all cell lines tested, but they also contain a fraction of regions (3%–5%) that display substantial structural variability (Fig. 1b, c; Supplementary information, Fig. S2a–c and Table S2).

RBP binding can be affected by the diverse cellular environments so such binding is expected to be dynamic across cell types. We re-analyzed available enhanced CLIP (eCLIP, all of the eCLIP data were downloaded from ENCODE^33^) data and indeed observed very different binding profiles for the same RBPs in different cell lines. For example, on average, anywhere between ~20% and ~60% of the binding sites are shared between K562 and HepG2 cells (Fig. 1d; Supplementary information, Fig. S2c). Importantly, we found these dynamic RBP binding sites are associated with the RNA structurally variable sites between the two cell types (Fig. 1b; Supplementary information, Fig. S2d). As an example, HNRNPM is known to preferentially bind poly-U sites with single-stranded structure.^34^ Indeed, the ratio of single- (icSHAPE score > 0.8) vs double-stranded (icSHAPE score < 0.2) regions for HNRNPM was 3.1:1 in HepG2 cells and was 3.8:1 in K562 cells, confirming HNRNPM’s preference for binding to single-stranded RNAs (ssRNAs). Notably, many HNRNPM binding sites overlapped with RNA structurally variable sites, and we detected reduced binding when these sites transitioned to a more double-stranded conformation in HepG2 cells (Fig. 1b), exemplified by the binding sites in the *LSS* and *FAH* transcripts in K562 cells (Fig. 1e; Supplementary information, Fig. S2e). Overall, these data support that RNA structure determines dynamic RBP binding interactions in diverse cellular conditions. An implication from these results is that the incorporation of in vivo RNA structural information into platforms that model and predict RBP bindings (and their changes across diverse cellular conditions) will enable more biologically relevant predictions.

### PrismNet accurately predicts cellular RBP binding by deep learning using in vivo RNA structural data

We constructed PrismNet, a deep neural network to accurately model and predict RBP binding, by integrating the in vivo RNA secondary structure profiles that we generated with the aggregated data for RBP binding sites. To ensure that the CLIP data sets used in our study are of high-quality and consistent, we downloaded the binding sites of 134 RBPs from the ENCODE project,^35^ as well as 56 RBPs from POSTAR,^36^ yielding a total of 168 RBPs (Supplementary information, Fig. S1). Note that the binding sites in these databases were generated using uniform pipelines, thereby eliminating differences from various tools and pipelines used by different labs in the original CLIP profiling studies. We also investigated use of different cutoffs for binding peak numbers, and found that using a set of the top 5000 ranking binding sites yields the highest extent of overlap ratio between replicates (Supplementary information, Fig. S3a). We therefore used the 5,000 most confident peaks from each CLIP experiment in training and testing a PrismNet model to help remove noise while still retaining a large number of binding peaks in the training dataset.

For each RBP with an available CLIP experiment, PrismNet trained a model that evaluates every nucleotide position within a binding site, and finally outputs a score for the whole binding site. Importantly, in contrast to previous methods that have only considered RNA sequences or which employ computationally predicted RNA structures, PrismNet was designed to simultaneously learn protein–RNA interaction determinants from both RNA sequence and in vivo structure data. As we show extensively below, this simultaneous learning approach ultimately proved vital for capturing the complex interplay between cell type-specific changes in structures and interactions in vivo.

**Fig. 2:**
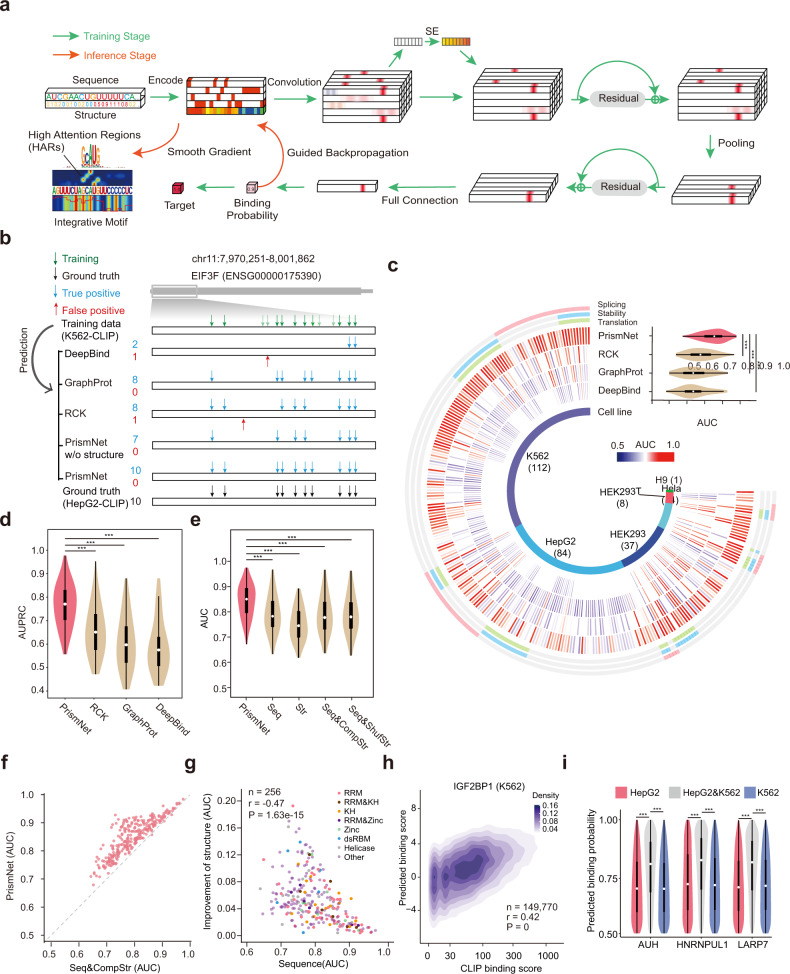
PrismNet predicts RBP binding in cellular conditions more accurately than methods which use only RNA sequence. **a** Model architecture of PrismNet. The input features include RNA sequence encoded in the 4-dimensional one-hot encoding, and the use of icSHAPE structural scores as the fifth-dimension. The neural network consists of multiple convolutional layers, squeeze-and-excitation (SE) networks, and residual blocks to capture the joint sequence-and-structural determinants of RBP binding. The green arrows indicate the data flow during network training, and the red arrows indicate the data flow during inference of HARs. **b** Predicted vs observed binding sites of IGF2BP1 on the *EIF3F* transcript. Green/black, observed binding sites in K562/HepG2 cells by eCLIP, used as the training/ground truth reference data; Blue and red indicate, respectively, true positive and false positive predictions in HepG2 cells, based on the models trained using K562 data. **c** “Circos and violin” plot of the respective and overall AUC scores of PrismNet vs other methods, including RCK, GraphProt, and DeepBind, for all 256 of the PrismNet models representing 168 human RBPs. ***P < 0.001 (one-sided paired *t*-test). **d** Violin plot of the overall AUPRC scores of PrismNet vs other methods for all 256 of the PrismNet models for 168 human RBPs. ***P < 0.001 (one-sided paired *t*-test). **e** Violin plot of the overall AUC scores of PrismNet models using different types of input data in all 256 PrismNet models of 168 human RBPs. ***P < 0.001 (one-sided paired *t*-test). **f** Scatter plot of AUC scores of PrismNet models using in vivo structures vs computationally predicted structures. Each dot represents an RBP. **g** Scatter plot of AUC improvements of PrismNet vs AUC scores of PrismNet models using only sequence information for 256 RBP models. Each dot represents an RBP model. RBPs are colored with their RNA-binding domains. RRM, RNA Recognition Motif; KH, K-Homology domain; Zinc, zinc finger domain; dRBM, double-stranded RNA binding motif. **h** Density map of binding scores of IGF2BP1 predicted using PrismNet vs the observed binding scores from eCLIP experiments in K562 cells. **i** Violin plot of PrismNet-predicted binding probabilities at the binding sites in K562 cells only, HepG2 cells only, or both. ***P < 0.001 (unpaired *t*-test).

The icSHAPE structure scores of each nucleotide in the same cell type of the CLIP experiment were encoded as a one-dimensional vector, together with the four-dimensional one-hot-encoded sequence as input (Fig. 2a). The PrismNet architecture uses a convolutional layer, a two-dimensional residual block^37^ and a one-dimensional residual block connected by max pooling to capture sequence and structural determinants spanning large distances in transcripts. A squeeze-and-excitation (SE) module^38^ is applied to adaptively recalibrate convolutional channels for learning channel-wise attention (Supplementary information, Fig. S3b). To mitigate potential overfitting of PrismNet, we added dropout^39^ layers after every residual block, and other regularizers including weight decay^40^ and early stopping in the training stage. Importantly, we also applied SmoothGrad^41^ to enable the enhanced saliency maps^42^ for the visualization and identification of high attention regions (HARs), which are predicted to be the exact locations of RBP binding nucleotides (Fig. 2a).

To demonstrate that PrismNet can accurately model the sequence and structural basis of RBP binding, we performed a proof-of-principle analysis with the RBP IGF2BP1, which plays important roles in regulating RNA stability and localized translation.^43^ Recently, eCLIP uncovered 46,226 IGF2BP1 binding sites in K562 cells.^11^ We trained PrismNet with this dataset and our icSHAPE data also in K562 cells. We then applied this model to predict the IGF2BP1 binding sites in HepG2 cells, using their corresponding HepG2 icSHAPE data. The predictions were then compared to the HepG2 eCLIP results from the same research group, as the ground truth dataset. According to eCLIP, the *EIF3F* transcript contains 14 IGF2BP1 binding sites in K562 cells, and 10 binding sites in HepG2 cells. We found that PrismNet correctly predicted all 10 binding sites within the EIF3F transcript in HepG2 cells with no false positives, by using the model trained in K562 cells. In contrast, DeepBind,^20^ correctly predicted only 2 of the 10 sites, and RCK^15^ and GraphProt^16^ correctly predicted 8 of the 10 sites (Fig. 2b). Interestingly, if we did not include RNA secondary structure data in the training of PrismNet, the sequence-only version correctly predicted only 7 of the 10 sites. This indicates that in vivo RNA structure information obviously improves the accuracy of PrismNet’s binding site prediction. This fact was frequently reiterated in our analyses of predictions for many other binding sites (Supplementary information, Fig. S3c).

We then systematically evaluated the prediction performance of PrismNet by using transcriptome-wide binding sites of all 168 RBPs, and comparing with other state-of-the-art computational methods, including RCK,^15^ GraphProt,^16^ and DeepBind^20^ (Supplementary information, Table S3). As mentioned above, DeepBind is a deep learning model to predict RBP binding sites based on RNA sequences alone; GraphProt models RBP binding sites based on sequence and predicted structure with graph-kernel features; RCK infers protein–RNA binding preferences using a *k*-mer-based model with RNA sequences and predicted structure. For every CLIP-seq dataset of an RBP, we split the binding sites into a training and a test set and use the same sets to benchmark all of the prediction methods. We observed that, overall, PrismNet achieved the highest prediction performance in terms of Area Under the receiver operating characteristic Curve (AUC) and Area Under the Precision-Recall Curve (AUPRC) (Fig. 2c, d; Supplementary information, Fig. S3d, e; *P* < 0.001, one-sided paired *t*-test). And some RBPs showed dramatic performance improvement by PrismNet (e.g., METAP2: PrismNet = 0.90 vs GraphPort = 0.65; AUH: PrismNet = 0.89 vs GraphPort = 0.63; DDX55: PrismNet = 0.88 vs GraphPort = 0.65).

### In vivo RNA secondary structure information is a critical input for accurate prediction of RBP binding

To dissect how RNA sequence and structural information contribute to the accurate predictions of PrismNet, we trained the PrismNet model using different combinations of input data: (i) sequence and experimentally-measured structure; (ii) sequence only; (iii) experimentally-measured structure only; (iv) sequence and structure predicted by RNAshapes;^44^ and (v) sequence and randomly generated structure (Supplementary information, Table S3). As expected, the model with sequence and experimentally-measured structure (i) as input significantly outperformed other models (Fig. 2e; Supplementary information, Fig. S3f). Notably, PrismNet achieved better performance on almost all RBPs over predictions based on sequence and computationally predicted structures (iv) (Fig. 2f). It is also interesting to note that PrismNet could fairly accurately predict protein–RNA binding sites using RNA secondary structure data only (iii), although the prediction accuracy was inferior to that only using sequence data only (ii).

Unexpectedly, we observed comparable prediction performance for the three models that use sequence data only (ii), sequence data with predicted structures (iv), and sequence data with randomly generated structures (v) (Fig. 2e; Supplementary information, Fig. S3f, g). A recent study showed that predicted structures did not improve the prediction performance of protein–RNA binding if the deep learning models were appropriately designed.^23^ This finding is consistent with our results: a good deep neural network can implicitly retrieve and use sequence-embedded RNA structure information, just like independent predictions. Incorporating separately predicted RNA structures thus cannot further improve the prediction (Supplementary information, Fig. S3h). However, it was surprising that training PrismNet with randomly generated RNA structures did not lead to a visible deterioration of prediction performance, implicating that PrismNet is robust to the noise in the input RNA structural data (Fig. 2e; Supplementary information, Fig. S3f). In addition, we also found that PrismNet performance was robust when using different ratios for positive vs negative ratios with more negative samples (Supplementary information, Fig. S3i, tested positive vs negative ratios included 1:1, 1:2, 1:3, 1:5, and 1:10), but slightly dropped for more positive samples (Supplementary information, Fig. S3j, tested positive vs negative ratios included 1:1, 2:1, 3:1, and 10:1).

Interestingly, the prediction upon inclusion of the experimentally measured in vivo structural data was in general improved for the RBPs having relatively poor predictions using sequence-only models (Fig. 2g). The level of improvement by the provided RNA structural information was associated with the type of RNA binding domain in the RBPs (Fig. 2g; Supplementary information, Fig. S3k). On the one hand, RBPs containing ssRNA-binding domains, such as the RNA recognition motif (RRM, e.g., HNRNPC and FUS) and the K homology (KH, e.g., HNRNPK and IGF2BP2) domains, were more dependent on RNA sequence for target recognition, and the prediction improvement from in vivo RNA structure was less substantial as compared to RBPs containing other domains (Supplementary information, Fig. S3k). On the other hand, RBPs containing a double-stranded RNA (dsRNA)-binding motif (dsRBM, e.g., SND1 and DGCR8) and helicase domains (e.g., DDX42 and DDX55) were the least accurate in RNA sequence-only predictions, and the improvement from RNA structural information was the most dramatic. These data reveal a trend indicating that RNA structure has a greater influence on the accuracy of PrismNet’s predictions for dsRNA-binding domains vs its predictions for ssRNA-binding domains.

We also noted that the predicted binding probability from PrismNet correlated with the binding affinity determined from CLIP experiments, as shown for different RBPs in K562 cells (Pearson correlation coefficient = 0.42 for IGF2BP1, *P* = 0) (Fig. 2h; Supplementary information, Fig. S3l, m). Although we only used “1/0” labels to denote the binding and non-binding events in the training dataset, PrismNet apparently learned a quantitative model for RBP binding from the big data of sequence, structure, and protein–RNA interaction. Unexpectedly, cell type-specific binding sites (Fig. 1d) generally had lower predicted binding scores compared to common binding sites (Fig. 2i, *P* = 0 for unpaired *t*-test). It therefore bears emphasis that PrismNet’s ability to predict dynamic RBP bindings with intermediate affinity should make it a useful tool for identifying such cell type-specific bindings.

Note that we have deployed all of the PrismNet models into a queryable service for RBP binding predictions online (http://prismnet.zhanglab.net/). This website should greatly facilitate access to the PrismNet models and our results, including the binding sites for both the PrismNet and experimental CLIP data for all 168 RBPs, as well as associated RNA structure information for six human cell lines.

### PrismNet predictions reveal putative regulators in post-transcriptional regulation events

Given PrismNet’s capacity as an accurate quantitative prediction tool for studying RBP binding, we next asked whether the predicted binding affinity for RBPs has any obvious relationship(s) with RBP-mediated post-transcriptional regulation. Many of the surveyed RBPs are splicing factors. To investigate the potential concordance between predicted binding sites and alternative splicing, we examined SRSF1, a splicing factor that functions in both constitutive and alternative pre-mRNA splicing.^45^ There are over 142,507 SRSF1 binding sites in HepG2 cells, detected by eCLIP.^11^ We trained PrismNet with this dataset and the HepG2 RNA structural data. PrismNet was then able to predict about 193,584 and 182,886 binding sites in HEK293T and K562 cells, respectively. 133,865 (~69%) of the binding sites in HEK293T cells were shared in K562 cells (or ~73% of the bindings sites in K562 cells were shared in HEK293T cells), suggesting a cell-type-specific binding pattern for SRSF1 (Supplementary information, Fig. S4a).

**Fig. 3:**
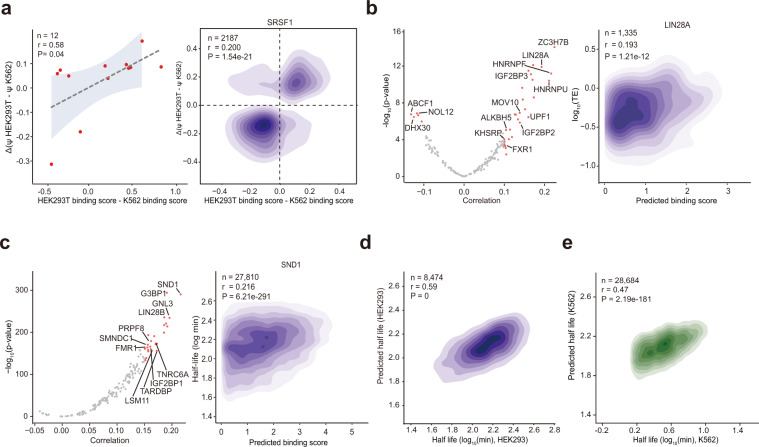
The quantitative predictions about RBP binding from PrismNet correlate with known splicing, translation, and degradation associated regulation events. **a** Pearson correlation of PrismNet-predicted differential SRSF1 binding and alternative splicing (difference of the percent-splice-in (PSI) values, STAR Methods) between HEK293T and K562 cells. Left, experimentally derived alternative splicing scores of 12 exons; Right, transcriptome-wide RNA-seq data. **b** Pearson correlation between PrismNet-predicted RBP binding and translational efficiency in HepG2 cells. Left, distribution of correlation coefficients of all RBPs with significance scores. Marked RBPs are known translation regulators. Right, density plot of the predicted LIN28A binding scores vs translation efficiencies for the target transcripts. **c** Pearson correlation between PrismNet-predicted RBP binding and RNA degradation in HEK293 cells. Left, distribution of correlation coefficients of all RBPs with significance scores. Marked RBPs are known RNA degradation regulators. Right, density plot of the predicted SND1 binding scores vs half-lives of the target transcripts. **d** Pearson correlation of experimentally measured and predicted half-lives in HEK293 cells. A machine learning model was trained for regression of the experimentally measured half-life, using the PrismNet-predicted binding scores for 168 proteins as the input. **e** Pearson correlation of experimentally measured and predicted half-lives in K562 cells. The prediction was based on the model trained in HEK293 cells, but employed the binding scores for the 168 proteins predicted by PrismNet in K562 cells as input.

To determine if dynamic SRSF1 binding sites correlate with alternative splicing, we examined the splicing levels of 12 exons that contain cell-type-specific binding sites in their 5′ splice sites (Supplementary information, Table S4). Indeed, we observed a positive correlation between the differential affinity of the binding sites and differential inclusion levels of these exons in K562 vs HEK293T cells (Pearson correlation coefficient = 0.58, *P* = 0.04; Fig. 3a, left). Given that the SRSF1 protein has been experimentally demonstrated to regulate RNA alternative splicing,^45^ our data support the hypothesis that SRSF1 binding may functionally contribute to exon inclusion during RNA processing.^46^ We further used RNA-seq data to test all the exons with cell-type-specific SRSF1 binding sites in their 5′ splice sites, and found that the differential affinity of the binding sites was modestly correlated with the differential splicing scores of the exons (Pearson correlation coefficient = 0.200, *P* < 1.54e-21; Fig. 3a, right).

In addition to alternative splicing, RBPs are also essential regulators of translation and degradation.^2,47^ We explored potential associations between the predicted binding sites and translation efficiency^48^ as well as RNA half-life^49^ for all surveyed proteins. Our analysis recovered many RBPs known to regulate translation efficiency, including the well-studied IGF2BP proteins,^43^ FXR1^50^ and LIN28A^51^ (Fig. 3b). Focusing on LIN28A, a well-known RBP that promotes translation,^51^ we found a positive correlation between the binding score predicted by PrismNet and the translation efficiency of the target transcripts (Pearson correlation coefficient = 0.193, Fig. 3b; Supplementary information, Fig. S4b). Moreover, we also tested many proteins that stabilize RNA, such as LIN28B,^52^ FMR1^53^ and SND1.^54^ We looked into transcripts with predicted binding sites of SND1, a protein that is known to regulate RNA half-life,^54^ and found that those transcripts indeed showed increased stability (Fig. 3c; Supplementary information, Fig. S4c).

For each protein, we intersected the two sets of binding sites from CLIP results and PrismNet predictions and split them into three groups: (a) the overlap between the CLIP results and PrismNet predictions (Common); (b) the set only predicted by PrismNet (PrismNet-specific); (c) the set only in the CLIP results (CLIP-specific). We then compared the correlations between binding affinity and RNA half-life for the three groups, respectively (Supplementary information, Fig. S4d, e). The Pearson correlation coefficients for the “Common” and the “PrismNet-specific” sets were greater than those for the “CLIP-specific” set, suggesting that binding sites from PrismNet predictions may be more biologically informative compared to raw CLIP data.

Furthermore, we constructed a Gradient Boosting Tree to predict RNA half-life using all PrsimNet predicted RBP bindings in HEK293T cells. This model was based on all RBPs, and achieved a much higher correlation between the predicted and experimental RNA half-life^49^ (Pearson correlation coefficient = 0.59, *P* = 0, Fig. 3d) than when using the individual proteins. Excitingly, we observed only a slight reduction in the correlation when we applied this model directly to K562 cells^55^ (Pearson correlation coefficient = 0.47, *P* = 2.19e-181, Fig. 3e), indicating how PrismNet predicted RBP bindings have the potential to be harnessed to generate accurate predictions of post-transcriptional regulation events.

This analysis also identified many putative novel regulators of RNA translation and half-life. For example, we found that ALKBH5 (an m^6^A eraser) may promote RNA translation, consistent with previously reported data about how excessively deposited m^6^A can reduce translation efficiency^56^ (Supplementary information, Fig. S4f). SMNDC1, an alternative splicing regulator, may promote RNA stability, consistent with a previous finding showing that alternative splicing could be coupled with nonsense-mediated mRNA decay^57^ (Supplementary information, Fig. S4g). These data collectively support that PrismNet, and especially its quantitative predictions of binding affinity, can aid studies of post-transcriptional gene regulation, including the prioritization of candidate post-transcriptional regulators and targets for further experimental investigations.

### PrismNet generates informative predictions about how structural alterations will affect RBP binding

Saliency maps are innovative tools developed in the computer vision field for visual attention retrieval.^41^ To characterize the binding preferences of RBPs, we developed a computational framework to capture the sequence and structural signatures of each binding site using a saliency map. The regions in each site that are important for RBP binding manifested as HARs in the saliency map, with the quantification of the contribution of every position to the binding. We used 20nt sliding windows to scan for HARs and iteratively merged two HARs if they have at least 1nt overlap. The lengths of the vast majority of HARs are within 20–40nt (Supplementary information, Fig. S5a). As a proof-of-concept, we visualized the HARs of a splicing regulator, Rbfox2. The highest attention regions (red, Supplementary information, Fig. S5b) indicate that a change at these positions, in sequence or structure, will result in the most dramatic changes in Rbfox2 binding probability. Our maps clearly revealed that HARs were enriched for “GCAUG” within single-stranded structures, consistent with previous studies of Rbfox2.^58^

HARs represent a theoretical model that quantifies the sequence and structural contributions of a nucleotide to RBP binding. It thus is important to separately assess their roles by experiments. To date, experimental validations of binding motifs have mainly considered how a sequence mutation affects RBP binding,^20,59^ so here we focused on how a structural change may also influence RBP binding beyond sequence alone.

**Fig. 4:**
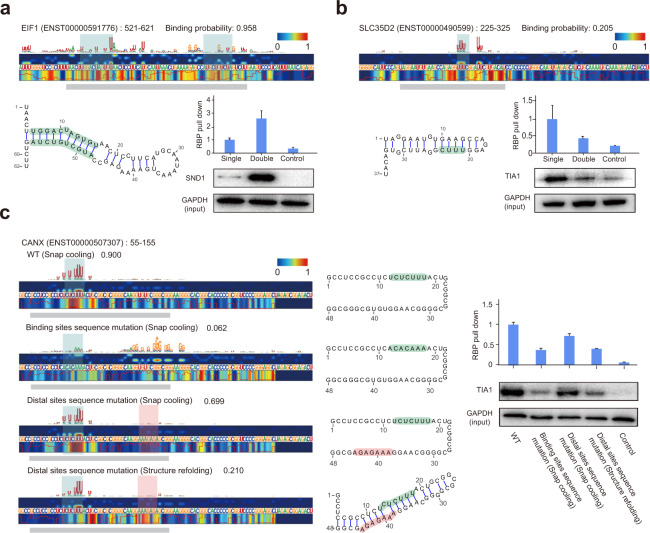
Validation of PrismNet-predicted effects of RNA sequence mutations and structure changes on RBP binding. **a** Experimental validation of SND1 binding on a site of the *EIF1* transcript (*EIF1*:521–621). Top, The saliency map showing the *EIF1* site in a double-stranded conformation, predicted to be bound by SND1. The two heatmap tracks display the response of the model at each nucleotide position, with red color showing HARs (upper track, sequence response; bottom track, structure response). The sequence logos on the top show the sequence patterns of high attention patterns. The red lines at the bottom represent icSHAPE scores. Gray bar, region of a synthesized RNA fragment; Green background, duplex region. Bottom left, predicted in vitro secondary structural model; Bottom right, RNA pull-down for the synthesized RNA fragment in a different structural conformation (*n* = 3 replicates), a random sequence “ccaacucuaugucgacugccaacucuaugucgacug” was used as a control. **b** Experimental validation of TIA1 binding on a site from the *SLC35D2* transcript (*SLC35D2*:225–325). Top, The saliency map showing the *SLC35D2* site in a double-stranded conformation, predicted not to be bound by TIA1. Gray bar, region of a synthesized RNA fragment; Green background, TIA1-bindng sequence motif. Bottom left, Predicted in vitro secondary structural model; Bottom right, RNA pull-down for the synthesized RNA fragment in different structural conformation (*n* = 3 replicates). A random sequence “ccaacucuaugucgacugccaacucuaugucgacug” was used as control. **c** Experimental validation of TIA1 binding at a site from the *CANX* transcript (*CANX*:55–155). Left, saliency maps showing the site of the wildtype (WT) and sequence mutants in different structural conformations: (i) WT *CANX* in single-stranded conformation; (ii) HAR sequence mutation, single-stranded conformation; (iii) distal site sequence mutation, single-stranded conformation; (iv) distal site sequence mutation, double-stranded conformation. Gray, region of synthesized RNA fragments; Green background, TIA1-binding sequence motif; Pink background, a distal site. Middle, predicted in vitro secondary structural models; Right, RNA pull-down for the synthesized RNA fragments in various structural conformations (*n* = 3 replicates, for each RNA fragment). A random sequence “ccaacucuaugucgacugccaacucuaugucgacug” was used as control.

We first used different melting-and-folding treatments to perturb RNA structure without altering sequence. Briefly, for a given RBP, we selected PrismNet-predicted RNA binding sites that form hairpin structures (both in vivo and in vitro), with the HAR residing on the stem. We heat-denatured the RNA fragment of the binding site, and then either slow-cooled to allow refolding into the hairpin structure or fast-cooled to retain single-stranded conformation.^60,61^ For example, PrismNet predicted a double-stranded binding site for SND1 in the transcript encoding eukaryotic translation initiation factor 1 (EIF1) in human K562 cells (Fig. 4a; Supplementary information, Table S5). We found that SND1 showed stronger affinity to the slow-cooled vs fast-cooled RNA fragment, consistent with our earlier finding about the higher affinity of SND1 for the double-stranded conformation (Fig. 4a). Using this approach, we also validated the structural preferences of TIA1 for single-stranded conformation (Fig. 4b). In addition, we recently used PrismNet to predict a set of host RBPs that bind to the SARS-CoV-2 RNA genome and regulate viral infectivity in cells.^62^ We used the same melting-and-folding strategy, as well as mutagenesis of the binding sites, and validated the binding and structural preference of Interleukin enhancer-binding factor 3 (ILF3) for double-stranded RNA structure on SARS-CoV-2 viral RNAs. All these data validated that PrismNet accurately predicted the structural preference of different RBPs.

As an orthogonal approach to investigate the effect of structural context of HAR, we perturbed the structure by mutating a distal sequence outside of the binding nucleotides. TIA1 is known to bind a poly-U sequence motif,^63^ and PrismNet discovered that the motif must be in a single-stranded region for high affinity binding. We also synthesized a host sequence fragment from the *CANX* mRNA containing a TIA1 HAR (Supplementary information, Table S5). Mutating the poly-U motif disrupted TIA1 binding, even when the whole sequence was kept single-stranded by snap-cooling, confirming the contribution of the poly-U sequence to TIA1 binding. In contrast, a mutation at a distal site resulted in diminished TIA1 binding only under assay conditions that promote base-pairing with the poly-U sequence, further confirming that TIA1 preferentially binds single-stranded poly-U sequences (Fig. 4c). Overall, these results verify that PrismNet generates informative predictions about how structural alterations will affect RBP binding.

### Integrative motifs from PrismNet reveal mechanisms of RBP–RNA recognition

**Fig. 5:**
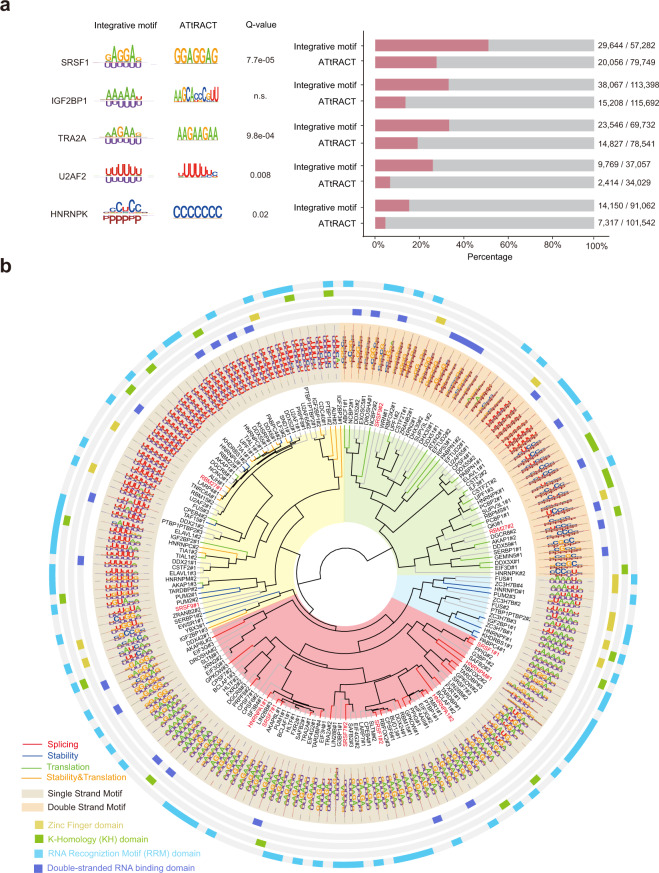
Integrative motifs derived from PrismNet are more specific in capturing transcriptome-wide binding site than canonical sequence motifs and cluster together with similar post-transcriptional regulatory functions. **a** Motifs of different RBPs. Left, the PrismNet integrative motifs and sequence motifs downloaded from the ATtRACT database for indicated RBPs. *Q*-values were calculated using TOMTOM^112^ for motif similarity between the sequence component of PrismNet integrative motifs and ATtRACT motifs. A PrismNet integrative motif comprises a sequence component (upper region of each logo) and a structural component (lower regions of each logo), where “U” stands for an unpaired nucleotide and “P” stands for a paired nucleotide. Right, paired with each RBP at the left; the number of true positives among all matched binding sites for the indicated RBP based on transcriptome motif scanning using the PrismNet integrative motifs and the ATtRACT motifs. True positives were determined by comparing with eCLIP experiments. **b** Hierarchical clustering of integrative motifs of human RBPs with available functional annotations.^113^ Colors represent functions in the inner tree, structural preferences in the circle of PrismNet integrative motifs, and RBDs in the outer circles. RBPs with red fonts are discussed in the main text. For each RBP, usually multiple motifs were defined (STAR Methods), which are indicated with the number after each RBP.

We aggregated and aligned all HARs to obtain the binding patterns for each RBP, defined as sequence-and-structure integrative motifs (integrative motifs). We calculated the integrative motifs for all the RBPs we had surveyed, and used a combined logo to denote a motif by adding a structural component to the nucleotide code (“P” for paired; “U” for unpaired; Supplementary information, Table S6).^64^ The sequence component of the integrative motifs that we discovered were highly consistent with the known sequence preference of these RBPs as documented in the ATtRACT database^65^ (e.g., GGA motif for SRSF1, poly-U motif for U2AF2), and with those derived from sequencing experiments by using *k*-mers enrichment^66^ or a recently reported method called mCross^67^ (Fig. 5a; Supplementary information, Fig. S5c, d).

In addition to sequence preferences, all RBPs that we surveyed exhibited preferences for specific RNA structures (Fig. 5a). PrismNet predicted that 69% of RBPs (116 out of 168 proteins) prefer single-stranded regions, consistent with previous studies.^17,68^ Importantly, with the structural constraints, integrative motifs had a much lower false positive rate in discovering true RBP binding sites compared to sequence-only motifs (Fig. 5a; Supplementary information, Fig. S5e). For example, 52% (29644/57282) and 25% (20056/79749) of the sites matched by the integrative motif and the sequence-only motif were covered by the experimental CLIP-seq data, respectively.

To systematically visualize and compare the sequence and structural specificities for each RBP, we performed hierarchical clustering of all associated integrative motifs. Interestingly, RBPs involved in the same RNA regulatory pathway were generally clustered together (Fig. 5b), which can be partially explained by their similar integrative motifs. For example, RBPs preferring the GA (AG/GAA) motifs in single-stranded RNAs were enriched for regulators of alternative splicing and were clustered together, including SRSF family proteins (e.g., SRSF1, SRSF7 and SRSF9) as well as some HNRNPs (e.g., HNRNPA1, HNRNPM). Many RBPs that bind to U-rich, A-rich or AU-rich motifs in single-stranded RNAs were associated with RNA stability and also clustered together (Fig. 5b), consistent with previous studies showing that the AU-rich elements (AREs) in 3′ UTRs target host mRNAs for rapid degradation.^50^

Some RBPs have multiple binding patterns, i.e., integrative motifs. For example, SRSF9 can bind to GUGGA in single-stranded structures and CCGGGA in double-stranded structures, and RBM27 can bind to polyU sequence in single-stranded structures and to UCCUC in double-stranded structures. These proteins generally contain multiple RBDs with different binding preferences that exert distinct regulatory roles.^68,69^ Overall, these observations suggest that integrative motifs can capture the determinants of RBP binding more accurately than canonical sequence motifs and agree with the domain composition and biological functions of the associated RBPs.

### HARs predicted by PrismNet represent evolutionarily conserved, functionally impactful sites

**Fig. 6:**
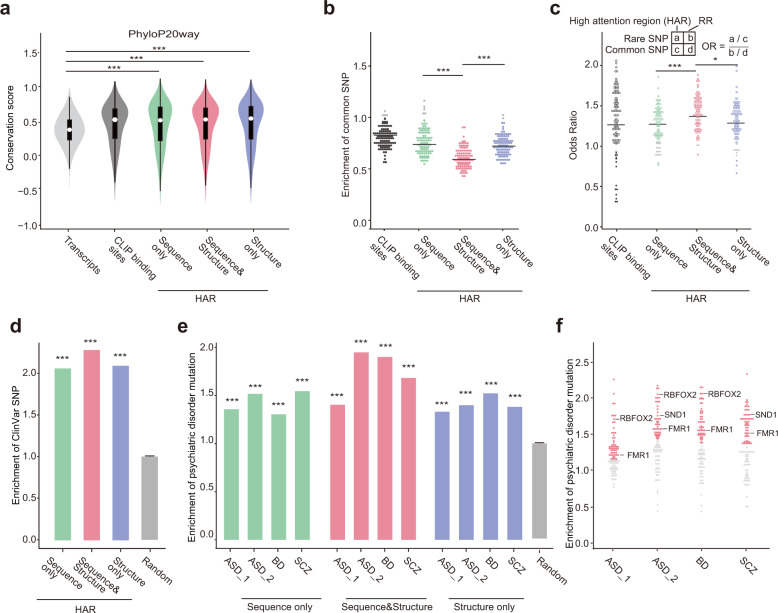
Conservation and enrichment of genomic variants in PrismNet-predicted HARs. **a** Distribution of PhyloP20way conservation scores of three groups of HARs (i.e., Sequence only, Sequence & Structure, and Structure only), with conservation scores for all CLIP binding sites and randomly sampled transcript regions as positive and negative controls, respectively. ***P < 0.001, one-sided unpaired *t*-test. **b** Enrichment of common SNPs (from dbSNP) in three groups of HARs and CLIP binding sites for every RBP, compared against randomly sampled transcript regions. ***P < 0.001, one-sided paired *t*-test. **c** Enrichment of rare SNVs (from dbSNP, Minor allele frequency (MAF) < 0.05%) relative to common SNVs in three groups of HARs and CLIP binding sites for every RBP. RR, randomly sampled transcript regions. *P < 0.05, **P < 0.01, ***P < 0.001, one-sided paired *t*-test. **d** Enrichment of ClinVar mutations in HARs, compared to randomly sampled transcript regions. ***P < 0.001, permutation test. **e**, **f** Enrichment of ASD, BD, and SCZ-associated variants among all HARs collectively (**e**) or HARs of every RBP respectively (**f**), compared to randomly sampled transcript regions. RBPs previously reported to be associated with psychiatric disorders are highlighted. ***P < 0.001, permutation test. The data of *ASD_1* are from An et al., 2018;^77^ the data of *ASD_2*, *BD* and *SCZ* are from Gandal et al., 2018.^76^ All HARs and CLIP-seq binding sites were here defined using data from K562 cells. In Fig. 6b, c, f, each dot represents an RBP. RBPs with HARs significantly depleted/enriched for genetic variants (including common and rare SNVs and disease-associated variants) are shown in color. Gray dots represent the non-significant RBPs. ***P < 0.001, permutation test for Fig. 6b, f, Fisher’s exact test for Fig. 6c.

An integrative motif of an RBP displays some degree of flexibility in sequence and/or structural contents at each position (Fig. 5b). Variations beyond the tolerance level may disrupt RBP binding and consequently lead to dysregulation. Indeed, using both the PhyloP and PhastCons scores from UCSC,^70,71^ we found that the HARs predicted by PrismNet are more evolutionarily conserved than the overall transcripts (*P* = 0, one-sided Student’s *t-*test; Fig. 6a; Supplementary information, Fig. S6a).

SNP density is also an indicator of evolutionary conservation.^72^ We analyzed the enrichment of SNPs at HARs vs the whole transcript. We initially separated the HARs into three groups according to their attention in primary sequence and/or secondary structure. Briefly, for each binding site, the regions with both high attention sequence and structure components in PrismNet belong to the “sequence & structure” group; the regions with only high attention sequence or structure component respectively belong to the “sequence only” or “structure only” group. We then calculated an odds ratio for each group to represent the enrichment of SNPs in HARs over background random positions for every RBP.

We first considered the common SNPs in the dbSNP database^73^ and found that all three groups showed a depletion of common SNPs for most RBPs (*P* < 0.05, permutation test, Fig. 6b). Furthermore, while the groups of “sequence only” and “structure only” showed comparable odds ratios, HARs in the group of “sequence & structure” were generally more depleted of SNPs (odds ratio: 0.60 (sequence & structure) vs 0.78 (sequence) or 0.75 (structure), *P* < 0.001, one-sides *t*-test). We confirmed these results using the common SNPs from the 1000 Genomes catalog^74^ (*P* < 0.05, permutation test, Supplementary information, Fig. S6b).

Next, we investigated the enrichment of rare variants, which are often deleterious in human populations.^75^ We found that the HARs of most RBPs are enriched for rare SNVs (*P* < 0.05, Fisher’s exact test; Fig. 6c; Supplementary information, Fig. S6c), suggesting that the variants within HARs tend to disrupt the functionality of the predicted binding sites. And again, the “sequence & structure” group showed higher enrichment than the other two groups (*P* < 0.05, one-sided Student’s *t-*test, Fig. 6c). To deconvolute conservation (and SNVs) based on functional sites vs RBP binding sites, we repeated the analysis by separating RBPs by function (splicing, translation, degradation). We found no obvious differences in any of these analysis between the three separate functional groups of RBPs (Supplementary information, Fig. S6d–f). Together, these data suggest that the HARs, particularly with both sequence and structure signatures, are evolutionary conserved and depleted of common SNPs, and tend to harbor deleterious rare mutations.

We thus further explored the relationship of variants in HARs with human disease. Indeed, genetic mutations deposited in the ClinVar database are enriched in HARs, especially in the “sequence & structure” group (Fig. 6d). Recently, large-scale neurogenomic studies have identified a large number of expression quantitative trait loci (eQTL) SNPs and de novo mutations associated with psychiatric disorders including autism spectrum disorder (ASD), schizophrenia (SCZ), and bipolar disorder (BD).^76,77^ In these studies, the effects of the identified variants were mostly explained by their ability to alter chromatin structure and TF binding. Interestingly, we found that these variants were also more enriched within the HARs (Fig. 6e). The odds ratios varied for RBPs within each disorder, with some specific RBPs exhibiting stronger enrichment (Fig. 6f). For example, RBFOX2, SND1, and FMR1 binding sites were frequently disrupted by ASD-associated variants, consistent with the roles of these proteins as revealed by previous studies.^78–80^ Notably, again, the “sequence & structure” HAR group showed relatively higher levels of enrichment for these psychiatric disorder-associated variants than the other HAR groups. These results support that analyzing mutations within the PrismNet HARs can informatively nominate putative RBP regulators and targets in complex human disorders.

### RNA structure-disruptive variants (RiboSNitches) are associated with dynamic RBP binding and disease

**Fig. 7:**
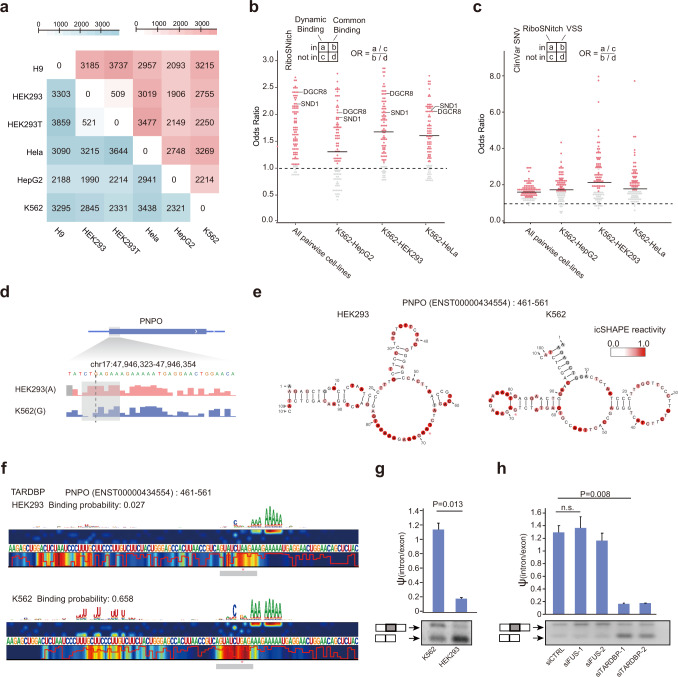
RiboSNitches are associated with RBP dynamic binding sites and human diseases. **a** The bottom-left triangle presents the number of putative riboSNitches detected in pairwise comparisons between two cell lines; the top-right triangle presents the number of these putative riboSNitches in the dynamic RBP binding sites predicted by PrismNet. **b** Enrichment of riboSNitches in dynamic RBP binding sites compared to common binding sites predicted by PrismNet for each RBP. “All pairwise cell lines” here refers to a pairwise comparisons among all cell lines. **c** Enrichment of riboSNitches relative to VSSs within HARs in human disease (from ClinVar). “All pairwise cell lines” here refers to a pairwise comparisons among all cell lines, while “VSS” refers to “variations of stable structure”, i.e., those genetic variations without RNA structure changes. In **b** and **c**, each dot represents an RBP, with significant enrichment shown in color (Fisher’s exact test). **d** Structural profiles of the icSHAPE scores around a riboSNitch in the *PNPO* transcript in HEK293 and K562 cells. **e** Structural models of a riboSNitch and flanking sequences in the *PNPO* transcript in HEK293 and K562 cells, predicted by RNAshapes with the corresponding icSHAPE scores as constraints. Red stars indicate the mutation sites. **f** Binding probabilities and saliency maps of TARDBP on the *PNPO* transcript in HEK293 and K562 cells. **g** Splicing of the *PNPO* transcript in K562 and HEK293 cells (*n* = 3 replicates). **h** Splicing of the *PNPO* transcript in WT and TARDBP knockdown K562 cells. FUS knockdown K562 cells were included as a negative control.

To further investigate the relationship between mutations in the predicted HARs and human disease, we focused on riboSNitches, a special class of SNPs or SNVs in which different alleles exhibit distinct foci RNA structures^81,82^ (Supplementary information, Fig. S7a). RiboSNitches are typically identified by allele-specific structural analysis in the same cells or cells from closely-related individuals.^81^ Here we developed a novel pipeline to uncover potential riboSNitches by comparing RNA structures at SNP sites among different alleles, and identified thousands of putative riboSNitches for each pair of cell lines (Fig. 7a; Supplementary information, Table S7). We intersected this dataset with the riboSNitches identified in a previous study in human lymphoblastoid cell lines^81^ and found a significant overlap (Supplementary information, Fig. S7b), confirming the validity of the dataset.

Notably, these riboSNitches are strongly associated with dynamic RBP binding (Fig. 7a, b). For every RBP in every pair of cell lines, we intersected the riboSNitches with the PrismNet-predicted dynamic binding sites. We found that most of these riboSNitches are located in a dynamic binding site of at least one RBP (Fig. 7a). And for most RBPs, their dynamic binding sites are enriched with riboSNitches, particularly for those RBPs with a preference for double-stranded structures (e.g., DGCR8, SND1) (Fig. 7b).

Many of the riboSNitches are also disease-associated variants from the ClinVar database.^83^ Strikingly, most of these disease-associated riboSNitches are located in dynamic RBP binding sites (Supplementary information, Fig. S7c and Table S7). Compared to variants that do not affect RNA structure, riboSNitches were enriched for disease-associated RBP-binding-disruptive mutations, both at the global level and at the level of individual RBPs (Fig. 7c). Note that this analysis only considered synonymous variants because we wanted to avoid impacts from possible protein truncations or frame shifts.

To clarify the regulatory relevance of riboSNitches in the context of disease association, we focused on a putative riboSNitch in the pyridoxamine 5′-phosphate oxidase (PNPO) gene for further validation. This riboSNitch corresponds to a synonymous G-to-A mutation (c.552 G > A; p.184 Leu > Leu) that has been associated with epilepsy, a severe neurological disorder.^84^ We found that HEK293 contains both alleles, whereas K562 contains the G allele (Supplementary information, Fig. S7d). The locus was indeed a riboSNitch, with very different RNA structures in the two cell lines (Fig. 7d, e). We then used PrismNet to scan each RBP for its binding probability at this local region and found that TARDBP had a much stronger binding affinity in K562 cells than in HEK293 cells (Fig. 7f; binding probability: 0.658 in K562 vs 0.028 in HEK293), consistent with the experimental CLIP-seq data (Supplementary information, Fig. S7e; K562: GSM2423707, GSM2423708; HEK293: DRX012638, DRX012639). TARDBP has been implicated in pre-mRNA splicing^85^ and we noticed that the *PNPO* exon containing this riboSNitch exhibited a dramatically reduced inclusion rate in HEK293 cells (Fig. 7g; *P* < 0.05, *t*-test). Interestingly, knockdown of TARDBP in K562 cells resulted in substantially reduced *PNPO* exon inclusion, in line with the phenotype of disrupted TARDBP binding in HEK293 cells (Fig. 7h; Supplementary information, Fig. S7f and Table S5). Collectively, these data indicate that riboSNitches, by disrupting the local RNA structure and/or directly altering RBP binding interactions, may help explain how disease-associated synonymous mutations can disrupt normal post-transcriptional regulation.

## Discussion

A substantial body of work has attempted to map RBP binding profiles to gain a more complete and mechanistic understanding of RNA regulation.^1–4^ Target recognition by RBPs has been found to be remarkably precise in vivo, yet this recognition apparently typically involves a limited primary RNA sequence space that is rich in low-complexity motifs.^29,86,87^ Binding site predictions with these low-complexity motifs inevitably contain many false positives. Local RNA structure of RBP binding sites is known to functionally impact the specificity of RBP recognition; however, previous predictive models have not included information for in vivo RNA structure. Here, we resolved these issues by generating a large dataset of RNA structural profiles across diverse mammalian cell lines. Our RNA structurome data show that RNA structure displays a significant amount of cell line-dependent variability, strongly suggesting its likely strong influence on the accuracy and relevance of model-based predictions of cell line-specific RBP binding. We implemented these insights into a deep neural network, PrismNet, constructed upon the large amount of cell line-specific RBP binding and RNA structurome data in vivo from matched cell lines. PrismNet learns protein–RNA interaction models and was therefore able to very accurately predict RBP binding in diverse cellular conditions.

Excitingly, we observed higher replicate correlations for binding sites from PrismNet predictions as compared with CLIP experiments, and also observed higher correlations between PrismNet predictions with experimentally determined post-transcriptional gene regulation events as compared with raw CLIP data. Both trends indicate that PrismNet-predicted output represents a refined set of putative binding sites, and suggest that use of PrismNet will give researchers a higher probability of identifying truly functionally impactful post-transcriptional regulators. Illustrating this application area, we showed how PrismNet output can be used for predicting RBPs likely to function in regulating translation efficiency and RNA half-life. Importantly, it bears emphasis that many RBPs are known to be multi-functional. Indeed, we also found that the SND1, LIN28B, and CPSF6 proteins apparently influence both translation efficiency per se and mRNA half-life. In addition, we observed that MOV10, IGF2BP1-3, and LIN28A were associated with translation efficiency, but the only functions reported to date for these proteins are related to mRNA half-life.^88–90^

The PrismNet model also allows us to dissect the relative contributions of RNA primary sequence and secondary structure. We verified that the change of RNA structure alone could affect RBP binding, once again highlighting RNA structure affects the dynamic regulation of RBP binding in vivo. Applying PrismNet models will support accurate predictions about how RBP bindings vary together with RNA structural changes in new physiological contexts. More broadly, our results also illustrate the promise of deep learning models for integrative data analysis and providing biological insights, rather than merely serving as black box classifiers.

By extracting features from a big dataset of binding sites with sequence and structural information, PrismNet learns a quantitative model of specificity determinants using the saliency map, in which the contribution of every position can be visualized. More importantly, with the model, PrismNet can predict mutations with severe consequences on RBP binding, and automatically dissect the perturbation effects resulting from the disruption of normal sequence and/or structure patterning. We identified and validated mutations in the PrismNet HARs  that are strongly deleterious in the human population, and are implicated with potentially important roles in complex human disorders.

Much work remains to be done to understand post-transcriptional regulation in different physiological contexts, particularly in disease-relevant conditions where mutations affect RNA structure and RBP binding. SNPs with allele-specific RNA structures, i.e., riboSNitches, are an emerging topic of great interest with ample opportunities for discovery. Although some candidates were found associated with diverse human disorders and phenotypes,^81^ we still do not understand the mechanistic basis of such correlations. We here comprehensively surveyed the associations of mutations with dynamic RBP binding and also human disease. Our study can support pursuit of the fascinating hypothesis that mutation–disorder associations may result from dysregulation of RBP binding.

Three recent publications have explored the use of allele-specific analyses with CLIP-seq data to identify SNPs that potentially modulate protein–RNA interactions.^67,91,92^ It is intriguing that these studies collectively revealed the apparently paradoxical finding that sometimes increased allele specific binding is not associated with gain of putative RBP motifs. Our study supports a related speculation: perhaps an increase in allele-specific binding is a consequence of improved structural context for RBP binding by riboSNitches. This idea could be explored in the future by embedding PrismNet within pipelines for allele-specific analyses of RBP bindings. This should support further refining of sequence-and-structure integrative motif characterization, and would likely facilitate elucidation of the specific pathologic influence(s) of dysregulated intrinsic sequences and/or structure, helping candidate prioritization for hypothesis-driven investigations on complex diseases. To this end, more RNA secondary structure profiles from disease-related tissue types will be valuable for dissecting the mechanisms of riboSNitches in a specific disease or phenotype context. Another important and further step would be to discover small molecule drugs that could potentially target RNA in a structure-specific manner.^93^ Together, the enhanced understanding of the regulatory mechanisms that govern the formation of RNA structure and its interaction with other biomolecules may pave the way to discovery of innovative therapeutic modalities for treating human disease.

## Materials and methods

### Cell culture and NAI-N3 modification in cells

Human HEK293 cells, HEK293T cells, K562 cells, HeLa cells, and HepG2 cells were bought from Cell Bank, Shanghai Institutes for Biological Sciences, Chinese Academy of Sciences. The mouse embryonic stem cell (mES) line E14T was a gift from the lab of Xiaohua Shen, Tsinghua University. Human embryonic stem cell (hES) line H9 was a gift from the lab of Kehkooi Kee, Tsinghua University. HeLa, HEK293, HEK293T, and HepG2 cells were cultured in DMEM medium supplemented with 10% FBS at 37 °C with 5% CO_2_ in 15 cm plates to gain ~80%–90% confluency. K562 cells were cultured in RPMI 1640 medium supplemented with 10% FBS at 37 °C with 5% CO_2_ in 15 cm plates to reach ~2 × 10^7^ cells. mES E14T cells were cultured as described previously.^94^ Briefly, mES E14T cells were grown at 37 °C with 5% CO_2_ in 10 cm plates in complete ESC culture medium (DMEM supplemented with 15% heat-inactivated FCS (fetal calf serum), 1% of nucleoside mix (Millipore), 0.1 mM nonessential amino acid, 2 mM Glutamax, 0.1 mM 2-mercaptoethanol and supplied with 1000 U/ml recombinant leukemia inhibitory factor). hES cells were cultured as described previously.^95^ Briefly, hES cells were grown at 37 °C with 5% CO_2_ in 10 cm plates in knockout serum replacer (KSR) plus bFGF medium (knockout DMEM, supplemented with 20% knockout serum replacer, 0.1 mM nonessential amino acids, 0.1 mM β-mercaptoethanol,1 mM L-glutamine, and 4 ng/mL recombinant human bFGF (R&D systems)).

All cells were collected at ∼80%–90% confluency and washed by PBS buffer. Cells were then treated with the icSHAPE reagent NAI-N_3_ with rotation at 37 °C for 5 min according to published protocol.^96^

### RNA extraction and rRNA depletion

RNA extraction and rRNA depletion for all human and mouse cells were performed as previously described^96^ with the following modifications. Cell pellets were heated at 50 °C for 5 min in 1 mL Trizol LS (Life Technologies) to improve the efficiency of RNA extraction. Cell lysates were purified by following the QIAGEN RNA cleanup protocol, and rRNA depletion was performed following the Dynabeads^TM^ mRNA DIRECT^TM^ kit (invitrogen) protocol. To improve the purification of polyA RNA, polyT probe selection was performed three rounds and validated using an Agilent 2100 Bioanalyzer.

### icSHAPE library construction and sequencing

icSHAPE sequencing libraries were constructed from rRNA-depleted RNA samples as previously described^96^ with the following modifications. To sequence the libraries on the HiSeq X system, we designed new oligonucleotides of the linker, reverse transcription (RT) primer, and P5/P7 amplification PCR primers as follows. Note that the experimental barcodes are for different libraries located on PCR primers, rather than on RT primers.

The linker of NAI-N_3_ modification samples:

/5rApp/AGATCGGAAGAGCACACGTCT/3ddC/;

the linker of DMSO control samples:

/5rApp/AGATCGGAAGAGCACACGTCT/3Biotin/;

RT primer:

/5phos/DDDNNNNNNNNNNAGATCGGAAGAGCGTCGTGGA/iSp18/GGATCC/iSp18/CAGACGTGTGC,

D = A/G/T and N = A/T/G/C are random barcodes to discriminate PCR duplicates;

P5-Solexa PCR primer:

5′-AATGATACGGCGACCACCGAGATCTACACTCTTTCCCTACACGACGCTCTTCC-3′;

P7-Solexa PCR primer:

5′-CAAGCAGAAGACGGCATACGAGATCGAGGCTGGTGACTGGAGTTCAGACGTGTGCTCTTCCGATCT-3′.

Here the underlined “CGAGGCTG” is the specific experimental barcode to distinguish different libraries. Libraries of all cell lines were sequenced on the HiSeq X system to ~200 million reads per replicate.

### Immunoblotting and RT-qPCR and PCR

Immunoblotting was used to verify RNA pull-down results, performed with antibodies for proteins SND1 (Proteintech), TIA1 (Proteintech) and GAPDH (Abcam). Elution samples of RNA pull-down were boiled at 95 °C for 10 min, followed by immunoblotting as previously described.^32^

RT-qPCR was performed to assess splicing differences with primers designed across two exons as previously described (Fig. 3a).^97^ PCR was also performed to measure the splicing differences of different isoforms (Fig. 7g, h). We also verified site specific mutations in the genome with PCR and sanger sequencing (Supplementary information, Fig. S7d).

All the primers used in this study are in Supplementary information, Tables S4 and S5.

### RNA pull-down

RNA pull-down assays were performed as previously described^98^ with the following modifications. For folded RNA, 1 μL (100 μM) RNA oligonucleotides were refolded by heating at 90 °C for 2 min, and then incubated at 30 °C for 5 min.^98^ For single-stranded RNA, to minimize the secondary structure, 1 μL (100 μM) RNA was heated to 90 °C for 2 min followed by snap cooling on ice.^43^ Human K562 cells (1 × 10^7^, for TIA1 and SND1 proteins) were lysed in lysis buffer (150 mM NaCl, 1 mM EDTA, 1% Triton X-100, 0.5 mM DTT, 50 mM pH 7.5 Tris-HCl, 0.1% sodium deoxycholate) with 10 μL phosphatase inhibitor cocktail (Promega), 2.5 μL SUPERase•In RNase Inhibitor (Life Technologies), then the cell lysate was incubated with the RNA probes. To inhibit RNA refolding during incubation, RNA (10 μL) and cell lysate (1 mL) were incubated at 4 °C for 30 min, then another 30 min incubation at 4 °C with 50 μL pre-washed MyOneC1 streptavidin beads added.^98^ The beads were washed with pre-cooled high salt buffer (50 mM Tris-HCl, pH 7.5, 1 M NaCl, 1% TRITON X-100) once and pre-cooled low salt buffer (50 mM Tris-HCl, pH 7.5, 150 mM NaCl, 5 mM EDTA) twice at 4 °C, with each wash for 2 min. Proteins were eluted (30 μL) with low salt buffer at 95 °C for 10 min. The eluted protein samples (8 μL) were quantified by immunoblotting with its specific antibody as previously described. Control samples were prepared identically to the lysate samples, with the exception that no RNA oligonucleotides were added (Fig. 4).

The wide type and mutation sequences of SND1-binding probes and TIA1-binding probes are in Supplementary information, Table S5.

### RNA interference

siRNAs against *TARDBP* and *FUS* (Supplementary information, Table S5) were transfected into K562 cells using Lipofectamine RNAiMAX (Life technologies, Carlsbad, CA) following the manufacturer’s instructions. Cells were grown at 37 °C with 5% CO2 for 48 h with siRNA transfection. The efficiency of siRNA knock down was validated by qPCR and (Supplementary information, Fig. S7f) and the splicing pattern of *PNPO* transcript was validated by PCR (Fig. 7h; Supplementary information, Table S4).

### icSHAPE score calculation and quality control

To process icSHAPE sequencing data and calculate icSHAPE structure scores, we used icSHAPE-pipe, which is an updated version of the original icSHAPE bioinformatics pipeline.^31,96^ In brief, raw reads were first collapsed to remove PCR duplicates and trimmed to remove adapters. Then, the processed reads were mapped to the human (hg38) and mouse (mm10) genomes, respectively, using STAR^99^ with the default parameters^31^ (Supplementary information, Table S1). icSHAPE scores were then calculated using icSHAPE-pipe. To ensure data quality, we only retained the icSHAPE scores for those nucleotides with read depth higher than 100.

To validate data quality, we calculated the Pearson correlation coefficients of replicate libraries for each cell line, as well as the agreement between our icSHAPE scores and the crystal structure of the human 18S rRNA using the AUC of the Receiver Operating Characteristic (ROC) curve line (Supplementary information, Fig. S1b–e).

Finally, the icSHAPE scores can be visualized at the following sites: http://prismnet.zhanglab.net/, with backups at: human, https://genome.ucsc.edu/s/sunlei/PrismNet_icSHAPE_hg38; mouse, https://genome.ucsc.edu/s/sunlei/PrismNet_icSHAPE_mm10.

### Structurally variable site analysis

To define a structurally variable site (Fig. 1b; Supplementary information, Fig. S2a, b), first we estimated the random noise of icSHAPE scores using replicates. We computed the scores using icSHAPE-pipe for each replicate, and calculated the L1 distance for each nucleotide between replicates. We aggregated all the L1 distances from the seven cell lines, which were used as the background distribution of the technical variations of icSHAPE scores. We defined the cutoff to be the top 5% of the L1 distances as the threshold of random noise (ΔS_noise_).

For each transcript, we split it into sliding windows (window size: 20nt, window step: 1nt), and compared the structural scores from two cell lines to identify structurally variable windows based on the two scores, as a significance score, as well as an average L1 distance score.

To calculate the significance score, we first defined a nucleotide with an icSHAPE score difference larger than ΔS_noise_ as a structurally variable nucleotide. We then counted the number of structurally variable nucleotides within each window. We assumed that this number follows a binomial distribution, and then calculated a significance score (*P* < 0.05) for each window to define structurally variable windows by Binomial test (see below). We also calculated the average L1 distance of the 20 nucleotides for each window. We aggregated these average distances from all pairs of cell lines, which were used as the distribution of window-wise structural variations. We defined the cutoff to be the top 10% of the average L1 distances, which was used as the second threshold (ΔL1) for the identification of structurally variable windows. Finally, only a window with an average L1 distance larger than ΔL1 and also with a significance score lower than 0.05 was defined as a structurally variable window. We merged all the overlapped windows into disjointed structurally variable sites for each transcript. All structurally variable sites are shown in Supplementary information, Table S2.

### RBP binding site data collection and processing

RBP binding sites from CLIP-seq were collected from POSTAR and ENCODE (eCLIP).^35,100^ In total, we collected 269 CLIP-seq datasets for 56 RBPs from POSTAR,^100,101^ as well as 392 eCLIP datasets for 134 RBPs from ENCODE.^35^ To ensure that the CLIP data sets used in our study are of high-quality and consistent, we downloaded the binding sites from the ENCODE project and a published database (POSTAR), in which the binding sites have been generated using a uniform pipeline; that is, we did not use the binding site data from the original publications (as called by different labs using various tools, pipelines, and parameters).

For a RBP with CLIP experiments in different cell lines, we constructed a PrismNet model for each cell line separately. For a RBP with multiple CLIP experiments in one cell line, we only chose one experiment of the highest quality (first filtered by the number of experiment replicates, then ranked by average sequencing depth among replicates). For any CLIP experiment of more than one replicate, we combined the overlap binding sites from all replicates. Specifically, we performed replicate normalizations, summing up and then merging them to use all the information of each replicate. The scores of each peak were normalized to [0, 1]. Overlapping binding peaks (at least 1nt) were then merged with the summed peak signals to yield a single peak.

We defined each resulted peak as a binding site. The length of a binding site was unified to 101 nt, where a region shorter than 101 nt was expanded from the middle to both sides and a longer region was cut from both sides to the middle. Finally, the top 5000 binding sites with the highest signals and at least 40% icSHAPE scores coverage were kept for the training and testing of PrismNet, as positive samples.

For each RBP, we also generated a negative sample of binding sites by randomly selecting 10,000 regions of 101 nt from the whole transcriptome, with at least 40% of icSHAPE score coverage and avoiding binding regions. For both the positive and negative samples, we randomly split them into training set and validation set by 4:1 to be used in PrismNet, respectively. To test the influence of using different ratios of positive vs negative reference sets, we tested this ratio with 1:1, 1:2, 1:5, and 1:10 to train PrismNet models and validated their performance. Finally, the positive vs negative ratio of 1:2 was used for model training.

### Definition of dynamic RBP binding sites and its association with structurally variable sites

For each RBP, we intersected and defined the set of common and dynamic RBP binding sites for a pair of cell lines, where overlapped binding sites was defined as common binding sites, and the other binding sites (i.e., only in one cell line) was defined as dynamic binding sites. To avoid technology noise between different CLIP protocols, we defined common and dynamic RBP binding sites between eCLIP datasets for the same RBP in different cell lines.^11^

Then, to analyze the relationship of each RBP dynamic binding sites with structurally variable sites, we only retained the binding sites with icSHAPE scores in both HepG2 and K562 cell lines. We calculated the number of overlapping regions between dynamic binding sites and structurally variable sites. Then, for a background distribution, we randomly selected the same number of regions from the transcripts with icSHAPE scores and counted the numbers of random regions intersecting with structurally variable sites. This process was repeated 1000 times. The *P* value of the enrichment was estimated by the rank of the number of overlapping regions between dynamic binding sites and structurally variable sites in the re-sampled distribution.

### Input data of PrismNet

For each 101 nt region, we labeled it as “1” if it is a positive sample or “0” if a negative one. We encoded the sequence with the one-hot encoding (A, C, G, U, 4-dimension), and combined it with the structure scores as the fifth dimension (icSHAPE values ranging from 0 to 1, 1-dimension). Missing icSHAPE scores (Null) were dubbed with “-1”. The labels and encodings were the input of PrismNet training (see below).

### PrismNet architecture

The input data of PrismNet are denoted as $\mathcal{S}$, including *N* samples, where each sample ${\text{s}}\in\mathcal{S}$ is an RNA region of length *L* = 101 nucleotides (nt). As mentioned above, each nucleotide corresponds to a sequence-and-structure vector of D = 5 dimensions. So, we encode $\mathcal{S}$ as a tensor $X \in {\Bbb R}^{N \times L \times D}$ and the binding probability of $Y \in {\Bbb R}^N$ is computed by:

$${\mathrm{Y = PrismNet}}(X)$$

PrismNet is then defined by the following set of functions.

$${\mathrm{PrismNet}}\left( X \right) = \sigma \left( {FC\left( {f_R\left( {f_S\left( {f_C(X)} \right)} \right)} \right)} \right)$$

σ is a *sigmoid* function, *FC* is a fully connection layer, and *f*_*C*_, *f*_*s*_, *f*_*R*_ are the formulation of the convolutional block, the squeeze-excitation block (*SE*)^102^ and the residual blocks defined in this work^37^ respectively, which are defined as follows:

$$f_C(x) = ReLU\left( {BN\left( {Conv_{K,P}\left( x \right)} \right)} \right)$$

$$f_S\left( x \right) = x \otimes SE(x)$$

$$f_R\left( x \right) = {\mathrm{ResidualBlock}}_{1}({\mathrm{AvgPool}}({\mathrm{ResidualBlock}}_{2}(x)))$$

In *f*_*C*_, *ReLU* is a rectifier linear unit activation function.^103^ *BN* denotes a batch normalization layer:^104^

$$ReLU\left( x \right) = {\mathrm{max}}\left( {0,x} \right)$$

$$\widehat x = BN\left( x \right) = \gamma \left( {\frac{{x - {\mathbf{E}}\left( x \right)}}{{\sqrt {{\mathbf{Var}}\left( x \right) + {\it{\epsilon }}} }}} \right) + \beta$$

*γ* and *β* and are the learnable parameter vectors of size *C* (the channel of the input tensor). **E**(*x*) and **Var**(*x*) are the mean and the variance over the mini-batches. *ϵ* is a value added to the denominator for numerical stability. During training, the batch normalization layer keeps running estimates of the computed mean and variance, which are then used for normalization during evaluation. The running estimates are kept with a default momentum (*m* = 0.1), finally, the normalized *x* is combined by the current estimation $\widehat x$ with the weight *m* and the previous estimated $\widehat x_p$ by the trained batches with the weight (1 − *m*). Mathematically, the update rule is:

$$\widehat {x}^{\prime} = (1 - m) \times \widehat x_p + m \times \widehat x$$

The *Conv*_*K,P*_ denotes the 2d-convolutional layers with a learnable kernel $K \in {\Bbb R}^{C \times l \times d}$ and a padding $P = \left( {p_l,p_d} \right)$, where $p_l = \frac{{l - 1}}{2},\,p_d = \frac{{d - 1}}{2}$, which makes the output shape of the convolution layer the same as the input. These kernels were designed to detect local sensitive regions per RBP binding, and resulted in the feature map $F = Conv_{K,P}\left( X \right)$, where

$$F_{n,c,i,j} = \mathop {\sum}\limits_{i = 1}^l {\mathop {\sum}\limits_{j = 1}^d {W_{c,i,j} \ast x_{n,i - p_l,\,j - p_d}} }$$

$$x_{n,i - p_l,\,j - p_d} = \left\{ {\begin{array}{*{20}{c}} {X_{n,i - p_l,\,j - p_d}} \\ 0 \end{array}\begin{array}{*{20}{c}} {{\mathrm{if}}\,p_l \,<\, i \,<\, l + p_l\,and\,p_d \,< \,j \,< \,d + p_d} \\ {{\mathrm{otherwise}}} \end{array}} \right.$$

In *f*_*S*_, the squeeze-excitation block, acts as a channel-wise self-attention to enable dynamic motif detection with weight recalibration. ⊗ refers to the channel-wise multiplication between the input and the learned *C*-dimensional vector by SE block. The SE block first squeezes the global sequence context information into a channel statistic by using a global average pooling function *f*_*sq*_, and then excite it to a set of channel weights scaled between 0 and 1 by applying a nonlinear transformation *f*_*ex*_ with two fully connection layers and a ReLU activation function defined as follows:

$${\mathrm{SE}}\left( x \right) = \sigma (f_{ex}(f_{sq}\left( x \right)))$$

$$\widehat z = f_{sq}\left( z \right) = \frac{1}{{L \times D}}\mathop {\sum}\limits_{i = 1}^L {\mathop {\sum}\limits_{j = 1}^D {z\left( {i,j} \right)} }$$

$$f_{ex}\left( {\widehat z} \right) = W_2\left( {ReLU\left( {W_1\widehat z} \right)} \right)$$

σ is the *sigmoid* function, $W_1 \in {\Bbb R}^{\frac{C}{r} \times C}$ and $W_2 \in {\Bbb R}^{C \times \frac{C}{r}}$ are the learnable parameters of the two fully connection layers, *r* is the dimension reduction ratio.

In *f*_*R*_, *ResidualBlock*_1_ and *ResidualBlock*_2_ are the residual blocks with 1d and 2d convolutional kernels, respectively.^37^ *ResidualBlock*_2_ aims at learning the sequence and structure combined patterns, and *ResidualBlock*_1_ is designed to learn the spatial context features where the precise binding sites localization are, and *AvgPool* is a average pooling layer to pool the 2d feature maps into the 1d vectors. The formulation of the residual block is defined below:

$$ResidualBlock_i(x) = ReLU(x + I(x))$$

$$I\left( x \right) = B_3\left( {ReLU\left( {B_2(ReLU(B_1(x)))} \right)} \right)$$

$$B_j(x) = BN\left( {Conv_j\left( x \right)} \right)$$

Where $i \in \{ 1,2\}$ denotes the 1- or 2-dimensional residual block in which the inner convolutional and batch normalization layer are also in the *i*-dimension.

The final binding probability *Y* is the output of a *sigmoid* layer which transform the predicted binding score of the *FC* layer output.

### Training of PrismNet

In total, we trained 256 PrismNet models for 168 human proteins (Supplementary information, Table S3), using the input data as described above (in the section “Input data of PrismNet”).

The training of a PrismNet model seeks to learn the parameters to minimize the loss function, which is the *L*_2_ regularization on all parameters and the binary cross entropy loss between the target labels *T* and predictions *Y*, over the training set.

$${\mathrm{Loss}}(T,\,Y) = - \frac{1}{N}\mathop {\sum}\limits_{i = 1}^N {\left[ {p_c \ast t_i \ast \log y_i + \left( {1 - t_i} \right) \ast \log \left( {1 - y_i} \right)} \right] + \lambda \left| {\Bbb W} \right|_2}$$

Where *t*_*i*_ is the target label; *y*_*i*_ is the predicted binding probability; ${\Bbb W}$ represents all the parameter of PrismNet; *N* is the batch size in training stage. The model parameters are optimized by the optimizer *Adam,*^105^ an extension to the stochastic gradient descent algorithm that performs a form of learning rate annealing with adaptive step-sizes in a computationally efficient way with little tuning on hyper-parameters. In addition, we adopted a warmup scheme^106^ with a linear scaling rule for adjusting learning rates to overcome optimization challenges early in training.

The *L*_2_ norm on all parameters acts as the weight decay term that reduces overfitting in a training model. To avoid overfitting, batch normalization and dropout are applied in the network, and early stopping is adopted in training. A batch normalization layer follows after each convolutional layer and a dropout layer follows after each residual block. Early stopping is applied to train PrismNet automatically, which stops the training procedure as soon as the performance on the validation dataset is no longer improved after *N*_*es*_ epochs.

Because PrismNet formulates the RBP binding prediction as a binary classification problem and the datasets are mostly imbalanced, area under the receiver operating characteristic curve (AUROC) was is selected as the measure, which performed better than the loss function. Only the parameter values of the best AUROC model on the validation data were saved.

### PrismNet hyperparameters

PrismNet hyperparameters were tuned experientially, through sequential exploration of the hyperparameter space over the training data set. The parameter tensor shape of each convolutional, pooling, and full connection layer is labeled in Supplementary information, Fig. S3a, and the settings of other parameters are as follows:

Dimension reduction ratio. The dimension reduction ratio of squeeze-excitation block was 2.

Dropout probability. Dropout followed after each residual block with the probabilities 0.5 and 0.3.

*L*_2_ norm penalty. We set the penalty weight *λ* *=* 1e-6.

Batch size. We used batch size 64 for all the experiments.

Learning rate. The base learning rate was 0.001.

Positive weight in Loss Function. The positive weight *p*_*c*_ in loss function was 2.

Training epochs. We trained each model with the maximal epoch 200. The training procedure was stopped when no improvements in the latest *N*_*es*_ = 20 epochs by early stopping.

Initial weights. We initialized weights with Kaiming initialization.^107^

Gradients clipping. The gradients were clipped by max norm below 5.0.

### Whole transcriptome RBP binding prediction and evaluation

PrismNet models of 168 human RBPs were then used to predict these RBPs’ binding sites in the whole transcriptome of all six human cell lines with their specific structures. All transcripts with icSHAPE scores were split into sliding windows (window size: 101nt, window step: 20nt) as the model input. The sliding windows with binding probability ≥ 0.5 in each cell line were defined as predicted binding sites.

For evaluation, we compared PrismNet prediction probabilities with the CLIP-seq binding sites in the validation set, using the AUROC measure.

To evaluate the quantitative predictions of PrismNet, we first transformed the RBP binding probability (*P*) into an RBP predicted binding score (*S*) using the following reverse sigmoid.

$$S = - ln\left(\frac{1}{P} - 1\right)$$

Then we calculated Pearson correlation coefficients between predicted binding score and the eCLIP peak signal (CLIP binding score) from CLIPper^11^ (Fig. 2h).

We predicted the binding probability of the common and dynamic RBP binding sites between HepG2 and K562 cells. Then we compared the binding probability of dynamic RBP binding sites (binding sites in K562 cell only or HepG2 cell only) and common RBP binding sites and calculated the *P* value using one-sided Student’s *t-*tests.

### Comparison of PrismNet with other methods

We compared the prediction performance of PrismNet with RCK, GraphProt, and DeepBind^16,20,108^ using AUROC and area under the precision-recall curve (AUPRC) measures. RCK, GraphProt, and DeepBind were trained with their default settings on the same training samples used for PrismNet, and were evaluated the same way as PrismNet.

We also compared the prediction performance of PrismNet with four PrismNet-variants respectively using the following inputs: only RNA sequences (input shape: 101 × 4), only icSHAPE RNA structural data (input shape: 101 × 1), RNA sequences and computationally predicted RNA structural data (input shape: 101 × 5), RNA sequences and shuffled icSHAPE RNA structural data (input shape: 101 × 5). All four of these PrismNet variants were trained and evaluated on 256 models representing 168 human RBPs the same way as for PrismNet.

To evaluate the model performance for datasets comprising different ratios of positive vs negative samples, we trained and validated the PrismNet model with a series of datasets, all having the same positive sites, but differing in the numbers of negative sites. Specifically, the examined ratios of positive vs negative sites included 1:1, 1:2, 1:3, 1:5, 1:10, 2:1, 3:1, and 10:1 to train different RBP model and to validate their performance with AUROC.

To further compare the binding sites of CLIP data and prediction by PrismNet and DeepBind (Fig. 2b; Supplementary information, Fig. S3c), we trained models using DeepBind, PrismNet, and an iteration of PrismNet which only employed RNA sequence information as input, in each case using IGF2BP1 binding sites in the K562 eCLIP data. The human genome was split into 1 M bins (without overlap) and we counted the number of binding sites in the K562 eCLIP data. We then applied these models to predict the binding sites for the IGF2BP1 protein across the HepG2 transcriptome by DeepBind, PrismNet, and PrismNet with only RNA sequence information, also using a sliding windows strategy. Finally, we examined the overlap of binding sites between the K562 experimental data and the predicted sites from the three prediction models (Fig. 2b; Supplementary information, Fig. S3c).

### Correlation analysis between PrismNet predicted RBP binding and splicing

To obtain the SRSF1 binding difference in HEK293T and K562 cells, the transcriptome-wide binding sites of SRSF1 were predicted using PrismNet and compared between the two cell lines (Fig. 3a).

First, the splicing differences of the 12 targeted exons with predicted differential SRSF1 binding between HEK293T and K562 cells were quantified using RT-qPCR. The test primers are in the Supplementary information, Table S4. For each possible splice event (e.g., an exon skip event), we calculated a percent-splice-in (PSI) value. This PSI is the ratio of normalized reads (indicating inclusion of a transcript element) over the total normalized reads for that event (both inclusion and exclusion).

Then for the transcriptome-wide splicing difference between HEK293T and K562, we built icSHAPE control libraries (DMSO-treated) in HEK293T and K562 cells to be used as common RNA-seq data. The sequencing reads were pre-processed the same way as the icSHAPE libraries (cutting adaptors and removing duplicates), and were aligned to the human genome (hg38) using Bowtie2 with default parameters.^109^ Then the mapped reads were used to calculate the splicing difference between HEK293T and K562 using SUPPA2.^110^ To exclude the difference of different types of alternative splicing events, we only considered the transcripts with skipped exons and SRSF1 binding difference in the 5′ of the exon. We then calculated Pearson correlation coefficients between the difference of PrismNet-predicted SRSF1 binding probability and the splicing difference between HEK293T and K562 cells.

### Correlation analysis between PrismNet predicted RBP binding score and RNA half-life and translation efficiency

The pre-calculated RNA half-life data from HEK293 cells^49^ and translation efficiency data from HepG2 cells^48^ were downloaded from previous studies. Briefly, the RNA half-life data were based on metabolic labeling of nascent RNA with the photoactivatable ribonucleoside analog 4-thiouridine, and the translation efficiency data were measured using Ribo-seq in the cell lines. The transcriptome-wide binding sites of all RBPs were predicted by PrismNet in the matched cell lines (HEK293, HepG2) based on the corresponding RNA structure data (Fig. 3b, c). For one RNA with several binding sites, we used the maximum of these binding probabilities to represent the RBP binding probability of this RNA. The RBP binding probability (*P*) is then transformed into RBP predicted binding score (*S*) by the following reverse sigmoid.

$$S = - ln\left(\frac{1}{P} - 1\right)$$

The Pearson correlation coefficients with *P* values between RBPs predicted binding score and RNA translation efficiency as well as half-life were calculated using R (https://www.r-project.org/).

We intersected the CLIP binding sites with PrismNet predicted binding sites in HEK293 and defined the “CLIP-specific”, “Common” and “PrismNet-specific” binding sites. For the RNA with “Common” and “PrismNet-specific” binding sites, we calculated the Pearson correlation coefficients with *P*-values between RBPs predicted binding score and RNA half-life. For the RNA with “CLIP-specific” binding sites, we calculated the Pearson correlation coefficients between RBPs CLIP binding score and RNA half-life. (Supplementary information, Fig. S4d, e).

To further analyze to relationship between RBP binding and RNA translation and stability regulation, we classified the RNAs into a Strong binding group (binding probability ≥ 0.8), a Medium binding group (0.5 ≤ binding probability < 0.8) and a Low or non-binding group (binding probability < 0.5). Then we calculated the cumulative distribution function of the translation efficiency or the half-life of different group RNAs. The *P* value was calculated by Student’s *t-*tests.

### RNA half-life prediction model

To further analyze the RNA half-life regulation by multiple RBPs, we constructed a Gradient Boosting Tree model to predict the RNA half-life by using the predicted binding scores of the 168 RBPs. We downloaded the RNA half-life datasets for HEK293 and K562 cells^49,55^ and predicted the 168 RBP prediction binding scores for their transcriptomes. Here using an L2 regularized Gradient Boosting Tree model, we trained and validated performance for HEK293 cells. Subsequently, we then used model trained in HEK293 cells to predict the RNA half-life in HepG2 cells by using the RBP prediction binding score in HepG2 cells. Then, we examined the Pearson correlation coefficients between the experimental half-life data and the Gradient Boosting Tree predicted half-life data.

### Saliency map

PrismNet used the saliency map as an “attention” strategy to highlight the nucleotide regions that are particularly influential to the decision whether the input is positive or negative. In the inference stage, a saliency map is generated by SmoothGrad,^41^ which could produce sharpened visual coherence of sequence and structure logo by averaging the gradient made from many tiny perturbations of a given input *x* through guided backpropagation^111^

When a sample $x \in {\Bbb R}^{L \times D}$ was fed into PrismNet, the binding score $y \in {\Bbb R}^1$ and the gradient *g*(*x*) with respect to *x* were computed simultaneously by,

$$g\left( x \right) = \frac{{\partial {\mathrm{PrismNet}}\left( x \right)}}{{\partial x}}$$

$$\widehat M\left( x \right) = x \odot \frac{1}{n}\mathop {\sum}\limits_1^n {g\left( {x + N\left( {0,\sigma ^2} \right)} \right)}$$

Where $\partial {\mathrm{PrismNet}}\left( x \right)$ represents the gradient of PrismNet. The size of $g\left( x \right) \in {\Bbb R}^{L \times D}$ is the same as the input *x*. In general, *g*(*x*) represents how much difference a tiny change in *x* would be made to the binding score and highlights key regions where motifs appear. For producing much sharper motif, *n* samples were generated by add a small gaussian noise $N\left( {0,\sigma ^2} \right)$ with standard deviation σ. $\odot$ denotes element-wise multiplication.

### High attention region finding

For each putative RBP binding site (in length of 101 nt), we split it into sliding windows (window size: 20 nt, window step: 1 nt), and calculated the average of both sequence and structure response signal from the smoothed saliency map.^41^ The sliding window with the highest response signal was defined as the high attention region (HAR). We used 20 nt sliding windows to scan for a HAR region and iteratively merged two HARs if they have at least 1nt overlap.

### Integrative motif construction

To minimize noise in the binding data, only the binding sites with a predicted binding probability > 0.8 were kept for the construction of integrative motifs of each RBP. The same sliding window strategy by which HAR were identified in the saliency map was applied with the following modification to obtain more precise motif containing regions: we only retained 20% of the sliding windows with the highest attention signals and merged the overlapped windows, resulting in a list of windows of high attention. Notably, some RBP binding sites might contain long HARs (for example, longer than 20 nt. Fig. 4a) or more than one separating HARs (Supplementary information, Fig. S5a). And, for an RBP with more than one PrismNet models in different cell types, we combined all PrismNet HARs.

We then enumerated the sequence-and-structure patterns of all *k*-mers (*k* =  6) in these windows. For each position, the sequence component was the nucleotide, while the structure component was labeled as “U “for unpaired nucleotide, representing an icSHAPE value ≥ 0.233 (0.233 is the median of all icSHAPE scores), and “P” for paired with an icSHAPE value < 0.233. The similar *k*-mers (at most one mismatch or shift for sequence, at most two mismatches or shifts for structure) were combined to build the sequence and structure integrative motif (Fig. 5a; Supplementary information, Fig. S5b, c), resulting a sequence PWM of 4 × 6 matrix (*p*_*ij*_, where *i* = 1, 2, 3, 4 corresponding to A, C, G, U, and *j* is site index, 1 ≤ *j* ≤ 6) and a structure PWM of 2 × 6 matrix (*p*_*rj*_, where *r* = 1, 2 corresponding to U, P, and *j* is site index, 1 ≤ j ≤ 6).

$$\mathop {\sum}\limits_{i = 1}^4 {p_{ij} = 1}$$

$$\mathop {\sum}\limits_{r = 1}^2 {p_{rj} = 1}$$

The weight (*W*) of each integrative motif to represent the motif proportion was defined by the *k*-mer frequency in target integrative motif for all *k*-mers. We further used TOMTOM^112^ to calculate the pairwise similarity of the motifs and combined similar ones (*P* value < 0.05).^112^ If there were no enriched motifs in the binding sites, the weights of motifs should be equal. For the top 10 highest weight motifs of each RBP, we compared the weight of each motif to 10% (i.e., assuming that the weights of the 10 motifs are equal) to calculate the motif enrichment and then filtered for the significantly enriched motifs (*P* value < 0.05, Fisher’s exact test). All the motifs we called are shown in Supplementary information, Table S6. In this way, we constructed 1–3 motifs for each RBP.

### Motif comparison and scanning

We used TOMTOM to compare our integrative motifs from PrismNet (only the sequence component of the motif) with other motifs in the ATtRACT database. Then, we filtered out the motifs with long length (> 10nt) and kept the ones with the most significant *Q* value (with the requirement to be < 0.05) as the corresponding motif to a PrismNet “integrative” motif. Specifically, for IGF2BP1, as the ATtRACT database only contains one motif, we still included this motif (AAGCACCCGUU) for further analysis although the matching *Q* value is not significant (Fig. 5a).

For a sequence motif of a RBP from the ATtRACT database, we used the FIMO tool from the MEME suite “fimo --verbosity 1 --text SRSF1.motif HepG2_hg38_Transcriptome_icSAHPE.fa > SRSF1_motif_site.txt” to scan the whole transcriptome using the associated PWM matrix for each motif and predicted a binding site for a motif match.^14,65^ (Fig. 5a; Supplementary information, Fig. S5d). The ratio of overlapped sites between the output of the scanning sites and the binding sites from eCLIP results of the RBP was then used for evaluating a given motif. To compare with motif scanning of the integrative motifs, we limited the analysis only to those transcripts that were covered by icSHAPE scores.

For a sequence-and-structure integrative motif, the motif scanning was also performed as FIMO algorithm description with the following modification (Fig. 5a; Supplementary information, Fig. S5d). Again, we classified the structure of each nucleotide into “paired” (denoted as P) and “unpaired” (denoted as U) according to their icSHAPE score (P: icSHAPE score < 0.233, U: icSHAPE score ≥ 0.233). A log-likelihood ratio score (*R*, log_10_ of the likelihood ratio of motif existing on specific position) for each integrative motif with respect to each position of sequence and structure in transcripts was calculated:

$$R = log_{10}\left( {\mathop {\prod}\limits_{j = 1}^6 {p_{ij}} \mathop {\coprod}\limits_{j = 1}^6 {p_{rj}} } \right)$$

*p*_*ij*_ is the probability of specific sequence nucleotide (*i* = 1, 2, 3, 4 corresponding to A, C, G, U) in position j. *p*_*rj*_ is the probability of specific structure pattern (*r* = 1, 2, corresponding to U, P) in position j.

To convert these scores into *P* values, six nucleotides sites covered by icSHAPE scores was randomly sampled from the whole transcriptome for 200,000 times and log-likelihood ratio scores of these sites were calculated as the random distribution.*P* value is then defined as the rank of *R* in the random distribution.

### Integrative motif clustering

For all significant (i.e., *P* value < 0.05) sequence and structure integrative motifs of the RBPs with available functional annotations (splicing, stability, or translation) and an RNA binding domain (RRM, KH domain, Zinc finger domain, and double-stranded RNA binding domain, *etc*.),^113^ we calculated the Euclidean distance between each pair of motif matrixes to measure their similarity:

$${\mathrm{Euclidean}}\,{\mathrm{distance}} = \sqrt {\mathop {\sum}\limits_{i = 1}^4 {\mathop {\sum}\limits_{j = 1}^6 {\left( {p_{ij}^1 - p_{ij}^2} \right)^2} } + \mathop {\sum}\limits_{r = 1}^2 {\mathop {\sum}\limits_{j = 1}^6 {\left( {p_{rj}^1 - p_{rj}^2} \right)} ^2} }$$

We then used hierarchical clustering for motif clustering.

### Conservation analysis of HARs

For each RBP, we used the sliding window strategy to identify HARs in the binding sites, for sequence HARs and structure HARs respectively. For each binding site, if the sequence and structure HARs are overlapped, they will be classified into sequence and structure combine groups (“Sequence & Structure”); otherwise, they will be distributed into sequence-only (“Sequence only”) or structure-only (“Structure only”) groups.

To quantify the sequence conservation of HARs, we calculated the conservation score distribution by using the PhastCon100 score^114^ and the PhyloP20way score^114^ for all HARs for comparing against the distribution of the conservation score in all transcripts (Fig. 6a; Supplementary information, Fig. S6a). We also included the sequence conservation of the experimental RBP CLIP sites as controls. Differences in sequence conservation scores between HARs and all transcripts were assessed using one-sided Student’s *t-*tests.

### Enrichment analysis of common SNPs in HARs

To analyze the SNP density of HARs (Fig. 6b; Supplementary information, Fig. S6b), we used the predefined common SNPs in the dbSNP database,^73^ and we defined common SNPs in the 1000 Genomes Catalog as those single nucleotide variant sites with major alternative allele frequency > 5%.^74^ In the whole transcriptome, we identified 2,909,183 common SNPs from dbSNP and 428,569 common SNPs from the 1000 Genomes Catalog.

We counted the number of common SNPs positioned within HARs. Then, for a background distribution, we randomly selected the same number of regions from the whole transcriptome and counted the numbers of common SNPs in these random regions. This process was repeated for 1000 times. The enrichment of common SNPs in HARs compared to the mean of random distribution was calculated as

$$Enrichment = \frac{{Number\,of\,Common\,SNP\,in\,HARs}}{{Average\,Number\,of\,Common\,SNP\,in\,random\,regions}}$$

The *P* value of the enrichment was estimated by the rank of *Number of Common SNP in HARs* in the random distribution. The enrichment of common SNPs in the experimental CLIP sites was also calculated as a positive control. The *P* value of difference of enrichment between different group HARs was calculated by one-sided Student’s *t-*tests.

### Enrichment analysis of rare SNVs in HARs

To measure the rare SNVs in HARs (Fig. 6c; Supplementary information, Fig. S6c), we used dbSNP^73^ and 1000 Genomes data.^74^ In the whole transcriptome, we identified 32,739,071 rare SNVs from dbSNP by excluding all common SNPs, and 6,181,362 rare SNVs for 1000 Genomes as single nucleotide variant sites with major alternative allele frequency values < 0.05%. As above, we also randomly selected the same number of regions from the whole transcriptome and counted the numbers of rare SNVs in these random regions. The enrichment (odds ratio) of rare SNVs in HARs compared to common SNVs (as above) was calculated as:

$$Odds\,ratio = \frac{{\frac{{Number\,of\,Rare\,SNVs\,in\,HARs}}{{Average\,Number\,of\,Rare\,SNVs\,in\,Random\,Regions}}}}{{\frac{{Number\,of\,Common\,SNPs\,in\,HARs}}{{Average\,Number\,of\,Common\,SNPs\,in\,Random\,Regions}}}}$$

The *P* value of the enrichment was estimated using Fisher’s exact test. The enrichment of rare SNVs in the experimental CLIP sites was also calculated as positive control. The *P* value of difference of enrichment (Odds ratio) between different group HARs was calculated by one-sided Student’s *t*-tests.

### Enrichment analysis of ClinVar variations in HARs

To analyze disease-associated variants in HARs (Fig. 6d), we used the ClinVar database.^83^ We basically repeated the process in the above two sections, but replaced common SNPs/rare SNVs with ClinVar variants. The enrichment of ClinVar variants in HARs comparing to the mean of random distribution was calculated as:

$$Enrichment = \frac{{Number\,of\,ClinVar\,variants\,in\,HARs}}{{Average\,Number\,of\,ClinVar\,variants\,in\,random\,regions}}$$

The *P* value of the enrichment was estimated by the rank of *Number of ClinVar variants in HARs* in the random distribution.

### Enrichment analysis of psychiatric disorder-associated mutations in HARs

To analyze psychiatric disorder-associated mutations in HARs (Fig. 6e, f), we used the datasets in two PsychENCODE Consortium studies that generated a panel of mutations associated with autism spectrum disorder (ASD), schizophrenia (SCZ), and bipolar disorder (BD)).^76,115^ We repeated the analysis process in the above sections, by using these psychiatric disorder-associated mutations. The enrichment of psychiatric disorder-associated mutations in HARs compared to the mean of a random distribution was calculated as:

$$Enrichment = \frac{{Number\,of\,disorder \hbox{-} associated\,mutations\,in\,HARs}}{{Avergae\,Number\,disorder \hbox{-} associated\,mutations\,in\,random\,regions}}$$

The *P* value of the enrichment was estimated by the rank of *Number of disorder-associated mutations in HARs* in the random distribution.

### RiboSNitch discovery

To define a putative riboSNitch, we first identified all single nucleotide variants (SNVs) with different alleles in any two human cell lines, using the RNA-seq data of the icSHAPE DMSO libraries.^96,116^ We quality controlled and mapped the sequencing reads using icSHAPE-pipe. For each pair of cell lines, we called genomic variants from the mapping results using Broad’s Genome Analysis Toolkit (GATK).^117^

For a SNV site, we defined it as a putative riboSNitch if it has different alleles in a pair of cell lines, and also has different RNA structures (i.e., there is a structurally variable site in the local region. See the section “Structurally variable site analysis” for the definition of a structurally variable site) (Fig. 7a; Supplementary information, Fig. S7a). All defined riboSNitches are presented in Supplementary information, Table S7. The other SNVs with different alleles but conserved structures between each pair of cell lines (i.e., SNVs not in a structurally variable site) were defined as variations of stable structure (VSSs).

We compared our list of riboSNitches with a dataset (Wan) previously identified among human lymphoblastoid cell lines (GM12878, GM12891, and GM12892),^81^ by intersecting the two sets in each pair of cell lines (Supplementary information, Fig. S7b). The enrichment (Odds ratio) of the riboSNitch overlapping was calculated as:

$${\mathrm{Odds}}\,{\mathrm{ratio}} = \frac{{\frac{{Number\,of\,riboSNitches\,overlapped\,with\,Wan}}{{Number\,of\,riboSNitches\,not\,overlapped\,with\,Wan}}}}{{\frac{{Number\,of\,SNVs\,overlapped\,with\,Wan}}{{Number\,of\,SNVs\,not\,overlapped\,with\,Wan}}}}$$

The *P* value of the enrichment was estimated using Fisher’s exact test.

### Enrichment analysis of riboSNitches in dynamic RBP binding sites

To analyze the relationship between riboSNitches and RBP binding (Fig. 7b), we first used PrismNet to predict transcriptome-wide RBP binding sites of all RBPs in the six human cell lines as described in the above section “Whole transcriptome RBP binding prediction and evaluation”. For each pair of cell lines, sliding windows with predicted binding probability ≥ 0.5 in both cell lines were defined as predicted common binding sites, while those with predicted binding probability ≥ 0.5 in only one cell line were defined as dynamic binding sites.

For each pair of cell lines, we intersected the riboSNitches and predicted dynamic binding sites and counted the number of overlapping riboSNitches (Fig. 7a). The enrichment (Odds ratio) of riboSNitches in dynamic binding regions compared to common binding regions was calculated as:

$${\mathrm{Odds}}\,{\mathrm{ratio}} = \frac{{\frac{{Number\,of\,riboSNitches\,in\,dynamic\,binding\,sites}}{{Number\,of\,riboSNitches\,not\,in\,dynamic\,binding\,sites}}}}{{\frac{{Number\,of\,riboSNitches\,in\,common\,binding\,sites}}{{Number\,of\,riboSNitches\,not\,in\,common\,binding\,sites}}}}$$

The *P* value of the enrichment was estimated using Fisher’s exact test.

### Analysis of relationship between riboSNitches and human disease

To analyze the relationship between riboSNitches and disease, we counted the number of riboSNitches being disease-associated variants from the ClinVar database (Supplementary information, Fig. S7c, left-bottom triangle and Table S7). As most of the riboSNitches are in dynamic RBP binding sites, we also counted the number of disease-associated riboSNitches located in dynamic RBP binding sites (Supplementary information, Fig. S7c, up-right triangle).

To analyze the potential role of riboSNitches in RBP binding and disease (Fig. 7c), we calculated the enrichment (odds ratio) of riboSNitchs in HARs associated with ClinVar variants, compared to variations of stable structure (VSSs):

$${\mathrm{Odds}}\,{\mathrm{ratio}} = \frac{{\frac{{Number\,of\,riboSNitches\,associated\,with\,ClinVar\,variants\,in\,HARs}}{{Number\,of\,riboSNitches\,not\,associated\,with\,ClinVar\,variants\,in\,HARs}}}}{{\frac{{number\,of\,VSSs\,associated\,with\,ClinVar\,variants\,in\,HARs}}{{number\,of\,VSSs\,not\,associated\,with\,ClinVar\,variants\,in\,HARs}}}}$$

The *P* value of the enrichment was estimated using Fisher’s exact test.

### TARDBP CLIP data analysis

To obtain TARDBP binding profiles in K562 and HEK293 cells (Supplementary information, Fig. S7e), we downloaded the CLIP data from a previous study (K562: GSM2423707, GSM2423708. HEK293:DRX012638, DRX012639).^36^ All sequenced reads were mapped to the human transcriptome (hg38) using bowtie2 with the following parameters (--local --non-deterministic –norc --local). We used *samtools* to remove potential duplicate reads that mapped to the same transcriptome location and used *IGV* for visualization and analysis.^118,119^

## Supplementary information

Figure S1

Figure S2

Figure S3

Figure S4

Figure S5

Figure S6

Figure S7

Table S1

Table S2

Table S3

Table S4

Table S5

Table S6

Table S7

## Data Availability

The accession number for the icSHAPE sequencing data of all cell lines reported in this paper is GSE145805. The scripts of PrismNet model architecture used in this project are available from github (https://github.com/kuixu/PrismNet), The scripts of all downstream analysis used in this project are available from github (https://github.com/huangwenze/PrismNet_analysis). A queryable service for RBP binding predictions online of all PrismNet models is available from the website (http://prismnet.zhanglab.net/).
