# Supplementary material for: Predicting dynamic cellular protein–RNA interactions by deep learning using in vivo RNA structures
Source: Cell Res. 2021 Feb 23;31(5):495–516. doi: 10.1038/s41422-021-00476-y (PMC7900654; doi:10.1038/s41422-021-00476-y)
Supplement: Supplementary file 1 — Figure S1 [file 41422_2021_476_MOESM1_ESM.pdf]

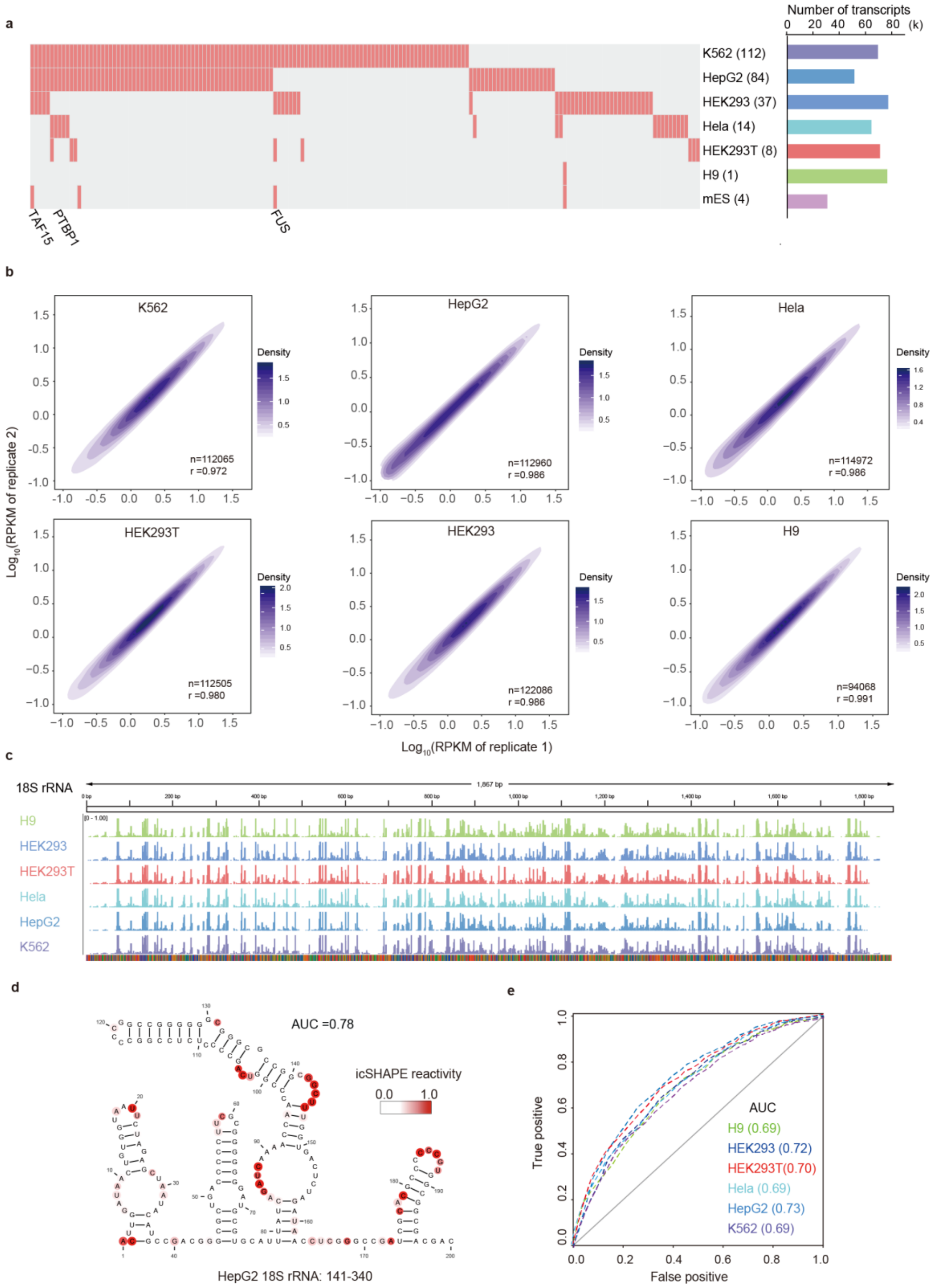


**Supplementary information, Fig. S1: Probing of RNA structuromes by icSHAPE in different cell lines.**

(**a**) Left: Heatmap of available RBP CLIP datasets in each cell line. RBPs with datasets in at least three cell lines were labeled. Middle: Cell lines with the number of available CLIP datasets. Right: The number of transcripts with secondary structure probed by icSHAPE

in each cell line.

(**b**) Pearson correlation of RNA expression (RPKM) between replicates of icSHAPE libraries.

(**c**) Tracks of icSHAPE scores of the human 18S ribosomal RNA.

(**d**) Structural model of 18S rRNA (141-340). The model was plotted with the *ViennaRNA* web service based on data from *RiboVision*[1], which is an experimental crystal structure from PDB (4V6X). Nucleotides are colored with icSHAPE scores from HepG2 cells.

(**e**) ROC curve plot of the agreement of icSHAPE scores from different cell lines with the reference 18S rRNA structure from *RiboVision*[1]. AUC scores for all ROC curves are included.

Reference:

1 Bernier, C. R. *et al.* RiboVision suite for visualization and analysis of ribosomes. *Faraday Discuss* **169**, 195-207 (2014).
