# Supplementary material for: Predicting dynamic cellular protein–RNA interactions by deep learning using in vivo RNA structures
Source: Cell Res. 2021 Feb 23;31(5):495–516. doi: 10.1038/s41422-021-00476-y (PMC7900654; doi:10.1038/s41422-021-00476-y)
Supplement: Supplementary file 2 — Figure S2 [file 41422_2021_476_MOESM2_ESM.pdf]

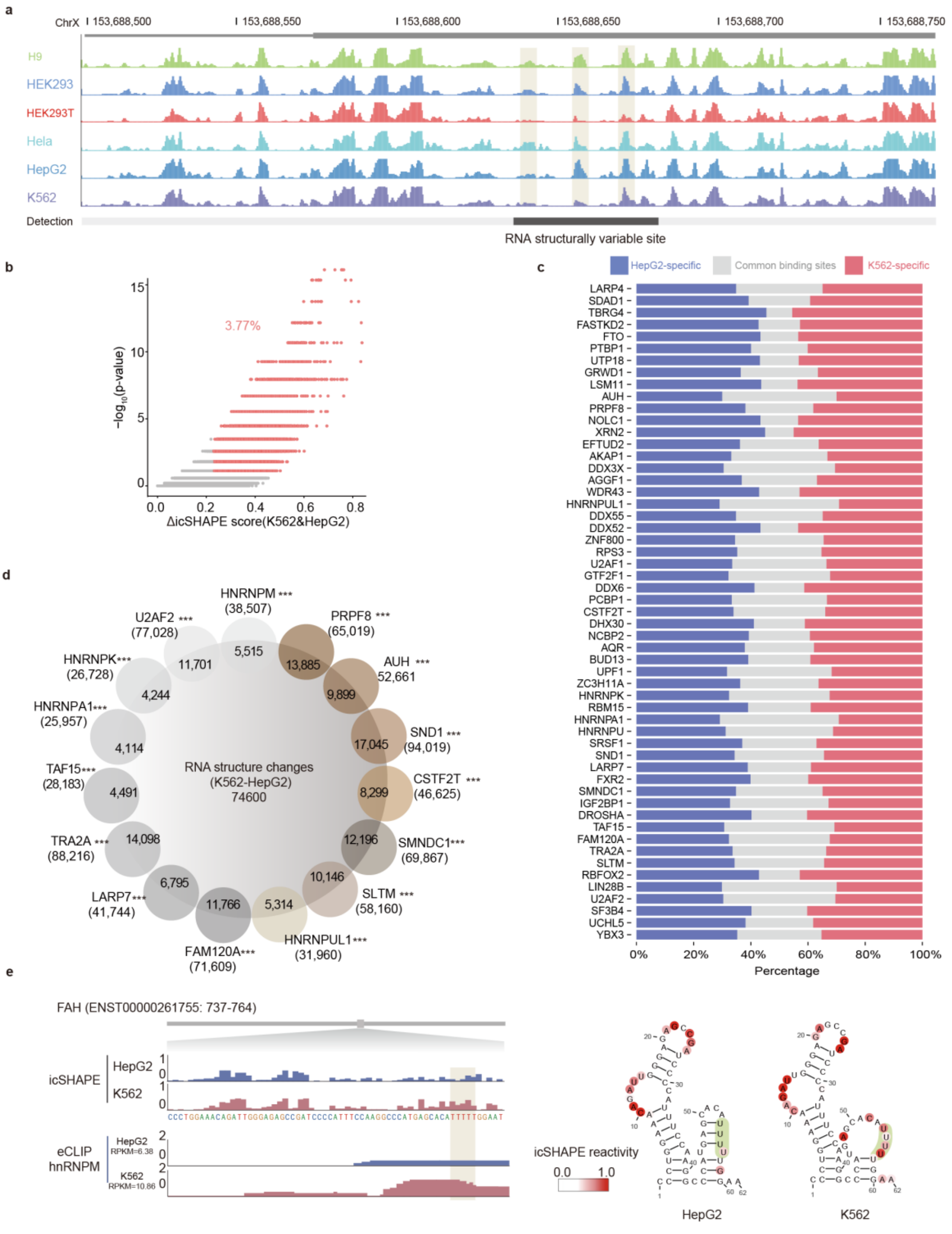

Supplementary information, Fig. S2: RNA secondary structurally variable sites, dynamic RBP binding profiles, and their associations.

(**a**) Tracks of icSHAPE scores at the human genome location chrx:153,688,450-153688850. The light gray background color labels a structurally variable site.

(**b**) Volcano plot shows the statistical significance versus magnitude of RNA structural

changes between HepG2 and K562 cells. The red plots are the identified structurally variable sites.

(**c**) Stacked bar plots of the percentage of dynamic (cell type-specific) and common RBP binding sites in two cell lines from eCLIP datasets: HepG2-specific (blue), K562-specific (red) and comm on sites (grey). The top 5000 ranking binding sites were used in both cell lines.

(**d**) The overlap between RNA secondary structurally variable sites (big central circle) and dynamic RBP binding sites (small surrounding circles) in HepG2 and K562 cells. The total numbers as well as the overlapped numbers are shown. *P* values were calculated by permutation test. $^{*}P < 0.05$; $^{**}P < 1 \times 10^{-3}$; $^{***}P < 1 \times 10^{-5}$. Note that this is not a Venn diagram, so that the overlaps among the RBP binding sites are not considered.

(**e**) RNA structural and HNRNPM binding profiles in HepG2 and K562 cell lines. Top: icSHAPE scores in the two cell lines for transcripts *FAH*. Middle: Binding site of HNRNPM on *FAH* (eCLIP). Bottom: RNA structural models of the HNRNPM binding sites on *FAH* in the two cell lines. Models are constructed by RNAshapes with icSHAPE score constraints. Green dashed lines indicate the HNRNPM binding motif.
