# Supplementary material for: Predicting dynamic cellular protein–RNA interactions by deep learning using in vivo RNA structures
Source: Cell Res. 2021 Feb 23;31(5):495–516. doi: 10.1038/s41422-021-00476-y (PMC7900654; doi:10.1038/s41422-021-00476-y)
Supplement: Supplementary file 3 — Figure S3 [file 41422_2021_476_MOESM3_ESM.pdf]

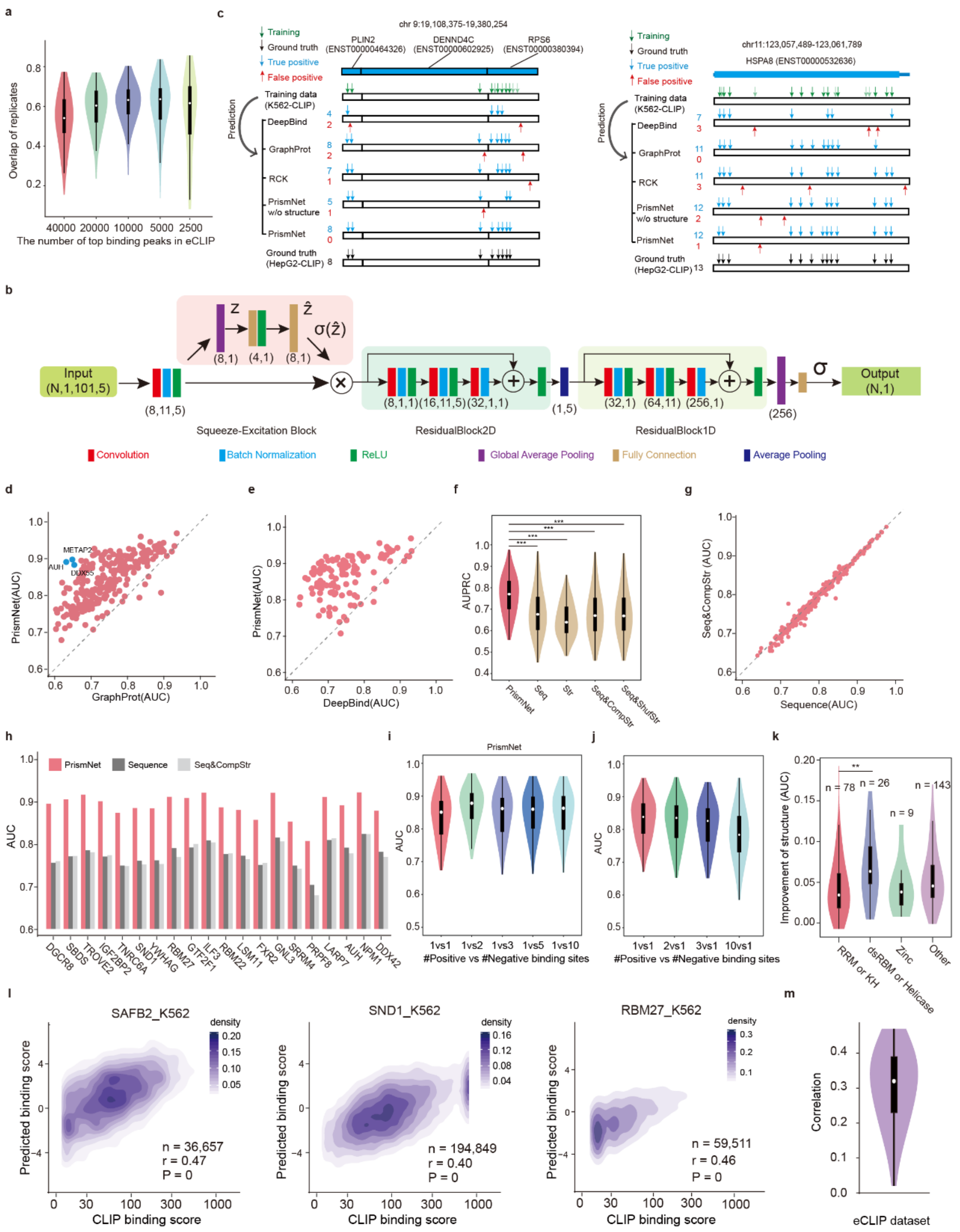

Supplementary information, Fig. S3: The architecture and prediction performance of PrismNet.

(**a**) Overlap ratio of CLIP binding sites between replicates, tested using different cutoff levels. The binding sites were ranked by binding score, and the top 5,000, 10,000, 20,000, and 40,000 binding sites were used to assess overlap between replicates.

(**b**) The architecture of the PrismNet model.

(**c**) Predicted versus observed binding sites of IGF2BP1 on the chr9:19,108,375-19,380,254 and chr11:123,057,489-123,061,789 regions. Green/black, observed binding sites in K562/HepG2 cells by eCLIP, used as the training dataset and the ground truth reference dataset, respectively. Blue/red, true/false positive predictions in HepG2 cells based on the models trained using K562 data.

(**d**, **e**) Scatter plot of AUC scores of the predictions by PrismNet versus GraphProt (**d**) and DeepBind (**e**). Each dot represents an RBP.

(**f**) Violin plot of the overall AUPRC scores of PrismNet models using different types of input data as indicated in all 256 PrismNet models of 168 human RBPs. ***P<0.001 (one-sided paired t-test).

(**g**) Scatter plot of AUC scores of the predictions by PrismNet with sequence and computationally-predicted structure versus with only sequence. Each dot represents an RBP.

(**h**) Bar plot of AUC scores of the predictions by PrismNet (red), PrismNet with only sequence (dark grey), and PrismNet with sequence and computationally-predicted RNA structure (light grey).

(**I, j**) AUC performance of PrismNet with different ratios of positive vs negative input data.

(**k**) Violin plot of AUC improvements of PrismNet predictions using *in vivo* structure for RBP groups with different RNA-binding domains. RRM: RNA Recognition Motif, KH: K-Homology domain, Zinc: Zinc Finger domain, dsRBM: double-stranded RNA binding motif. *$P < 0.05$; **$P < 0.01$; ***$P < 1 \times 10^{-3}$ (one-sided unpaired t-test).

(**l**) Density plot of the binding probability of SAFB2, SND1, and RBM27 predicted by PrismNet versus the observed binding scores from eCLIP in K562 cells.

(**m**) Violin plot of the Pearson correlation coefficients between PrismNet-predicted binding scores and CLIP binding scores from eCLIP for 49 RBPs.
