# Supplementary material for: Predicting dynamic cellular protein–RNA interactions by deep learning using in vivo RNA structures
Source: Cell Res. 2021 Feb 23;31(5):495–516. doi: 10.1038/s41422-021-00476-y (PMC7900654; doi:10.1038/s41422-021-00476-y)
Supplement: Supplementary file 4 — Figure S4 [file 41422_2021_476_MOESM4_ESM.pdf]

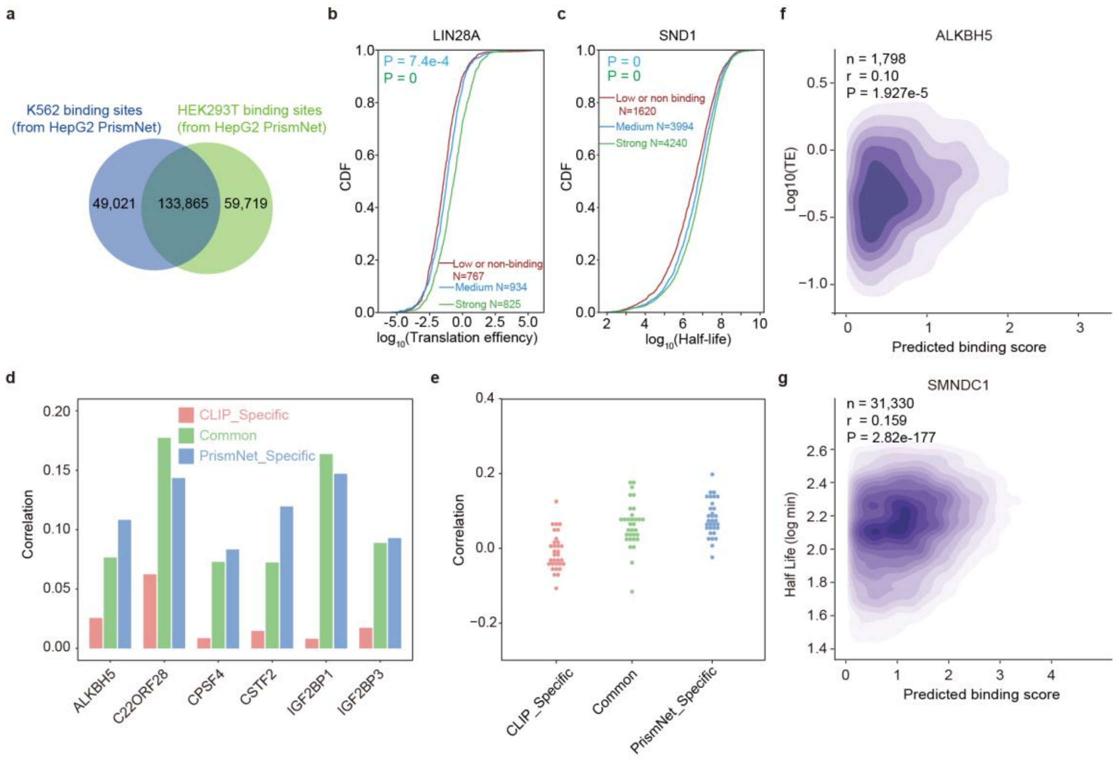

Supplementary information, Fig. S4: PrismNet-predicted differential RBP binding, and correlation of predicted quantitative RBP binding with target translation and degradation.

(**a**) Venn diagram of SRSF1 binding sites in HEK293T and K562 cells, predicted by the PrismNet model trained on the HepG2 SRSF1 eCLIP dataset.

(**b**) Cumulative distribution plots of transcript translation efficiency versus different levels of binding probability for RBP LIN28A: Strong binding (predicted binding probability ≥0.8), Medium binding (0.5≤ predicted binding probability <0.8), and Low or non-binding (predicted binding probability <0.5).

(**c**) Cumulative distribution plots of transcript half-life versus different level of binding probability of RBP SND1: Strong binding (predicted binding probability ≥0.8), Medium binding (0.5≤ predicted binding probability <0.8), and Low or non-binding (predicted binding probability <0.5).

(**d**) Bar plot of Pearson correlation coefficients between PrismNet-predicted binding score and RNA half-life values for PrismNet-specific predictions (blue), common bindings (green), and between CLIP binding score and RNA half-life values for CLIP-specific results (red). The pre-calculated RNA half-life data in HEK293 cells were downloaded from a previous

study[2].

(**e**) Scatter plot of Pearson correlation coefficients between binding score of each RBP and RNA half-life values for PrismNet-specific predictions (blue), common bindings (green), and CLIP-specific results (red). Each dot represents an RBP.

(**f**) Density plot of the PrismNet-predicted ALKBH5 binding scores versus translation efficiency of the target transcripts.

(**g**) Density plot of the PrismNet-predicted SMNDC1 binding scores versus half-lives of the target transcripts.

Reference:

2 Schueler, M. *et al.* Differential protein occupancy profiling of the mRNA transcriptome. *Genome Biol* **15**, R15 (2014).
