# Supplementary material for: Predicting dynamic cellular protein–RNA interactions by deep learning using in vivo RNA structures
Source: Cell Res. 2021 Feb 23;31(5):495–516. doi: 10.1038/s41422-021-00476-y (PMC7900654; doi:10.1038/s41422-021-00476-y)
Supplement: Supplementary file 5 — Figure S5 [file 41422_2021_476_MOESM5_ESM.pdf]

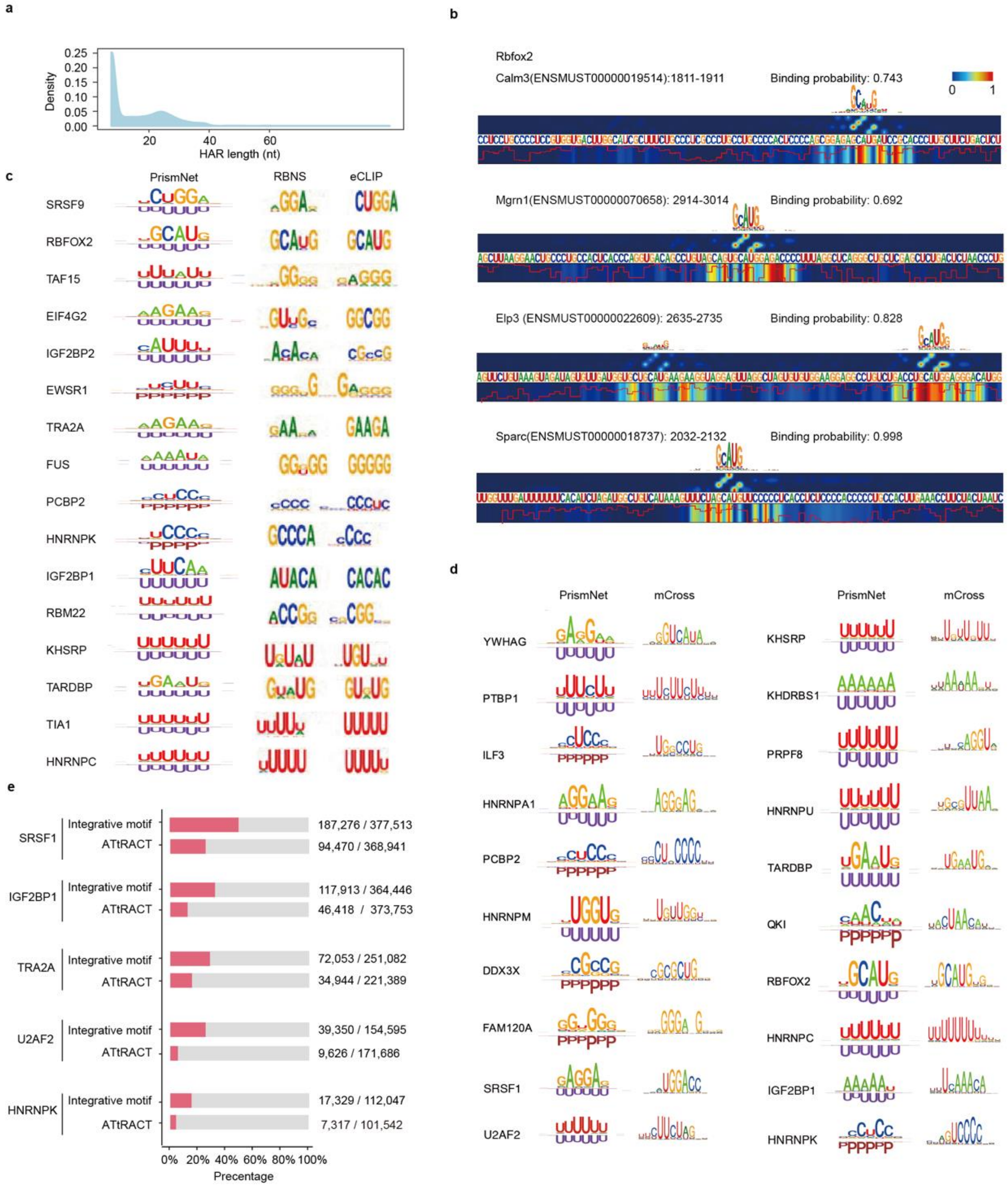

Supplementary information, Fig. S5: Saliency maps of PrismNet-predicted binding sites and Integrative motifs of different RBPs.

(**a**) Length distribution of HARs.

(**b**) Saliency maps of Rbfox2 binding sites derived from the mES PrismNet model. The heatmap tracks display the response of the model at each nucleotide, with red color showing high attention (upper: sequence response, bottom: structure response). The sequence logos on the top are shown to help to display high attention sequence component. The red line at bottom represents icSHAPE scores.

(**c**) Integrative motifs derived from PrismNet, compared with those provided in eCLIP and

RBNS.

(**d**) Integrative motifs derived from PrismNet, compared with those obtained by mCross.

(**e**) True positives and all matched binding sites on the transcriptome by motif scanning using the integrative and the ATtRACT motifs of different RBPs. True positives are determined by comparing with eCLIP experiments. Related to main Fig. 5a. Here we used a loose criterion in motif scanning to match more sites.
