# Supplementary material for: Predicting dynamic cellular protein–RNA interactions by deep learning using in vivo RNA structures
Source: Cell Res. 2021 Feb 23;31(5):495–516. doi: 10.1038/s41422-021-00476-y (PMC7900654; doi:10.1038/s41422-021-00476-y)
Supplement: Supplementary file 6 — Figure S6 [file 41422_2021_476_MOESM6_ESM.pdf]

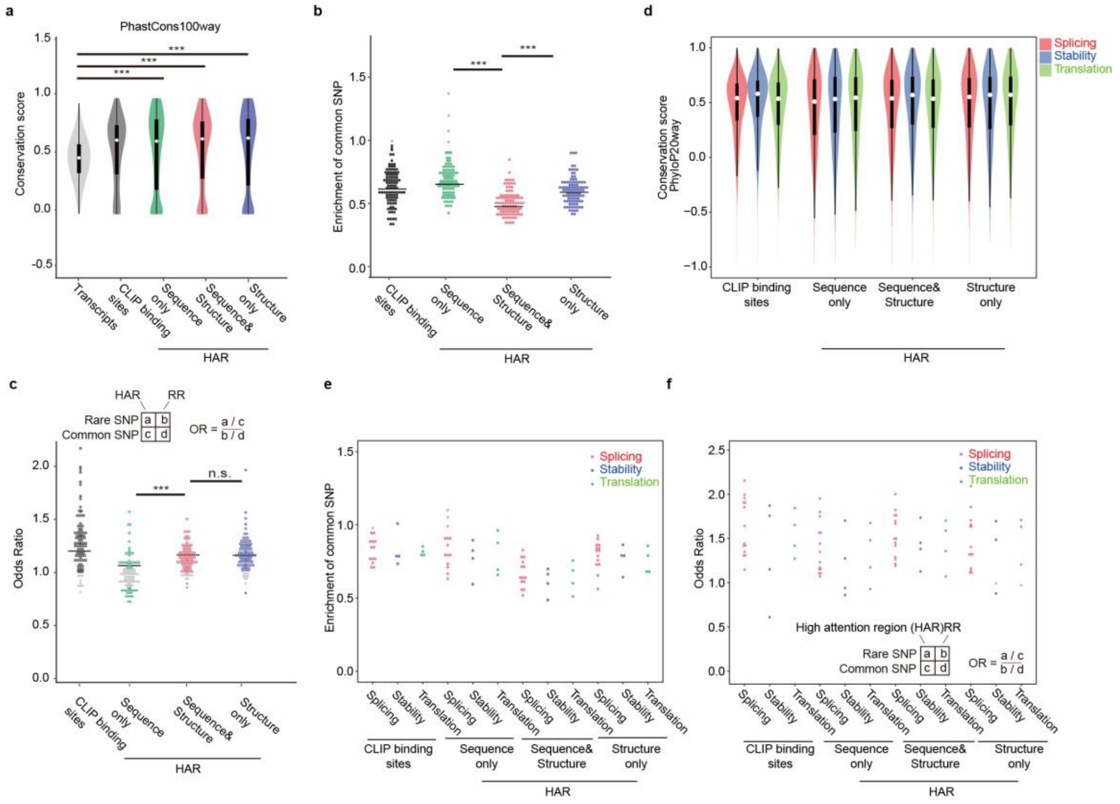

Supplementary information, Fig. S6: Conservation and enrichment of genomic variants in PrismNet-predicted high attention regions (HARs)

(**a**) Distribution of PhastCons100way conservation scores of HARs, with those of all binding sites from CLIP-seq experiments and random transcript regions as positive and negative controls. ****P*<0.001, one-sided unpaired t-test.

(**b**) Enrichment of common SNPs (from 1000 Genomes) in HARs and CLIP binding sites for every RBP comparing to random transcript regions. ****P*<0.001, one-sided paired t-test.

(**c**) Enrichment of rare SNVs (from 1000 Genomes, Minor allele frequency (MAF) < 0.05%) relative to common SNVs in HARs and CLIP binding sites for every RBP. n.s. not significant, ****P*<0.001, one-sided paired t-test.

(**d**) Distribution of PhyloP20way conservation scores of three groups of high attention regions (HARs) (*i.e.*, sequence only, sequence & structure, and structure only) for splicing associated RBPs (red), stability associated RBPs (blue), and translation associated RBPs (green), with those of random CLIP binding sites as positive controls. ****P*<0.001, one-sided unpaired t-test.

(**e**) Enrichment of common SNPs (from dbSNP) in three groups of HARs and CLIP binding sites for every RBP (split into splicing associated RBPs (red), stability associated RBPs (blue), and translation associated RBPs (green)) compared to random transcript regions.

(**f**)Enrichment of rare SNVs (from dbSNP, Minor allele frequency (MAF) < 0.05%) relative to common SNVs in three groups of HARs and CLIP binding sites for splicing associated RBPs (red), stability associated RBPs (blue), and translation associated RBPs (green).

All HARs and CLIP-seq binding sites are in K562 cells. In **b**, **c**, **e**, **and f**, each dot represents an RBP. RBPs with HARs significantly depleted/enriched of genomic variants are shown in color (*for P<0.05, permutation test or Fisher's exact test). Grey dots represent the insignificant RBPs. In **d**-**f**, we retained only RBPs with a single reported function. Splicing RBPs: "FUS, HNRNPK, HNRNPM, NONO, PRPF8, RBFOX2, RBM22, SRSF1, SRSF7, SRSF9, TRA2A, U2AF1, U2AF2"; Stability RBPs: "LIN28B, PUM2, TAF15, XRN2";Translation RBPs: "ATXN2, DDX3X, EIF4G2, IGF2BP2"; The annotation of RNA function is from the Yeo group[3].

Reference:

3 Van Nostrand, E. L. *et al.* A large-scale binding and functional map of human RNA-binding proteins. *Nature* **583**, 711-719 (2020).
