# Supplementary material for: Predicting dynamic cellular protein–RNA interactions by deep learning using in vivo RNA structures
Source: Cell Res. 2021 Feb 23;31(5):495–516. doi: 10.1038/s41422-021-00476-y (PMC7900654; doi:10.1038/s41422-021-00476-y)
Supplement: Supplementary file 7 — Figure S7 [file 41422_2021_476_MOESM7_ESM.pdf]

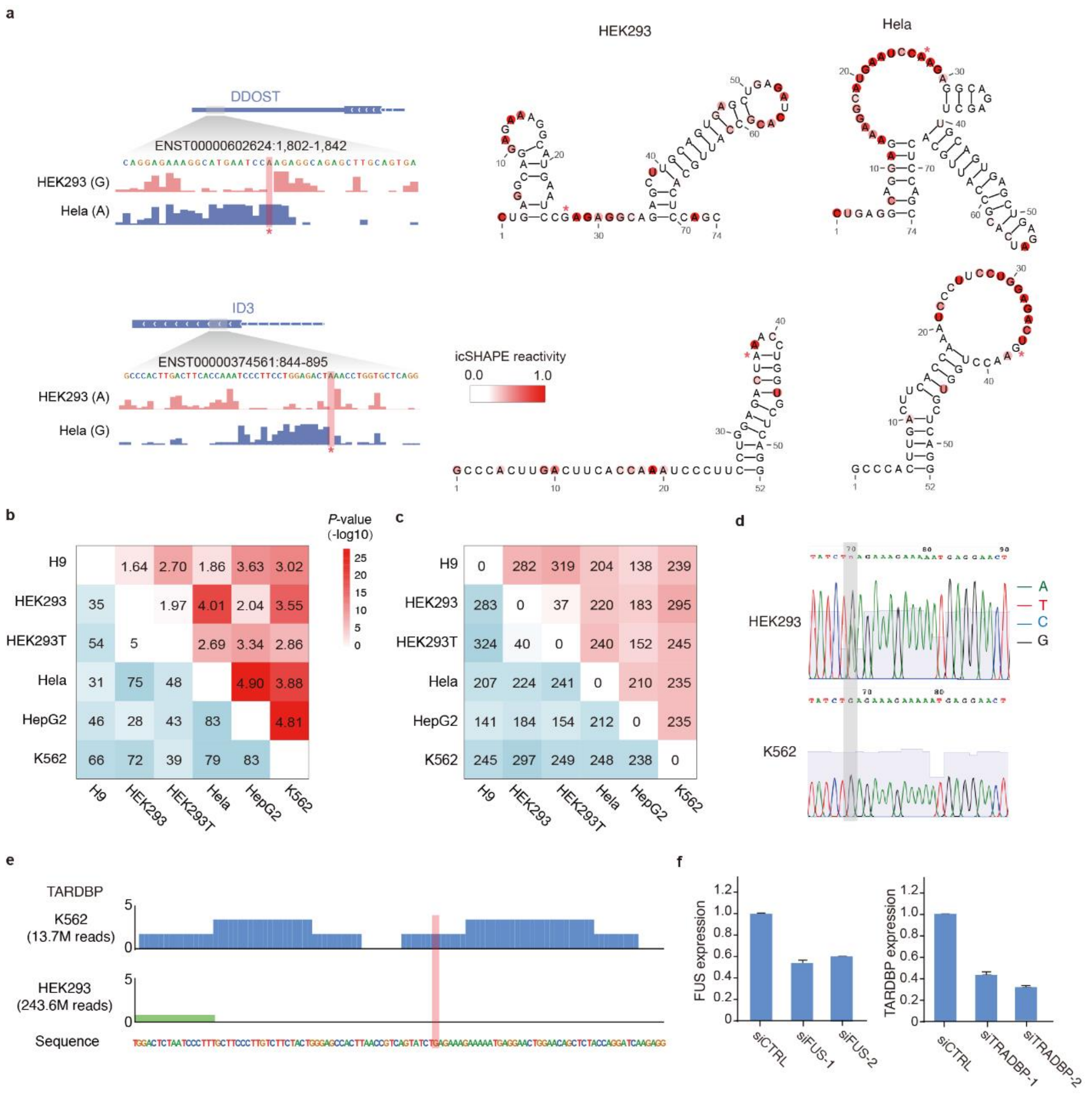

Supplementary information, Fig. S7: RiboSNitches between different cell lines and their association in RBP dynamic binding sites and human diseases.

(**a**) RiboSNitches in the *DDOST* and *ID3* transcripts. Left: Tracks of icSHAPE scores around the riboSNitches in HEK293 and HeLa cell lines. The dashed lines indicate the riboSNitch sites. Right: RNA structural models constructed by RNAshapes with icSHAPE score constraints. The red stars indicate the SNP sites in the structural model.

(**b**) The number (bottom-left triangle) and the odds ratio, enrichment significance (up-right triangle) of detected riboSNitches intersecting with the riboSNitches identified in human lymphoblastoid cell lines[4].

(**c**) The number of all riboSNitches corresponding to a SNV in ClinVar (bottom-left triangle) and those that are also in dynamic RBP binding sites predicted by PrismNet (up-right triangle).

(**d**) Validation of the riboSNitch alleles in the *PNPO* gene in HEK293 and K562 cells by Sanger sequencing.

(**e**) TARDBP binding tracks in K562 (eCLIP) and HEK293 (PARCLIP) in the *PNPO* transcript. The dashed line indicates the riboSNitch site.

(**f**) The knock down efficiency of FUS and TARDBP in K562 cells.

Reference:

4 Wan, Y. *et al.* Landscape and variation of RNA secondary structure across the human transcriptome. *Nature* **505**, 706-709 (2014).
